# Arousal Rules: An Empirical Investigation into the Aesthetic Experience of Cross-Modal Perception with Emotional Visual Music

**DOI:** 10.3389/fpsyg.2017.00440

**Published:** 2017-04-04

**Authors:** Irene Eunyoung Lee, Charles-Francois V. Latchoumane, Jaeseung Jeong

**Affiliations:** ^1^Communicative Interaction Lab, Graduate School of Culture Technology, Korea Advanced Institute of Science and TechnologyDaejeon, South Korea; ^2^Beat Connectome Lab, Sonic Arts & CultureYongin, South Korea; ^3^Center for Cognition and Sociality, Institute for Basic ScienceDaejeon, South Korea; ^4^Department of Bio and Brain Engineering, Korea Advanced Institute of Science and TechnologyDaejeon, South Korea

**Keywords:** visual music and emotion, aesthetic experience, auditory-visual perception, art and emotion, aesthetic perception, cross-modal integration, auditory-visual integration, music and emotion

## Abstract

Emotional visual music is a promising tool for the study of aesthetic perception in human psychology; however, the production of such stimuli and the mechanisms of auditory-visual emotion perception remain poorly understood. In Experiment 1, we suggested a literature-based, directive approach to emotional visual music design, and inspected the emotional meanings thereof using the self-rated psychometric and electroencephalographic (EEG) responses of the viewers. A two-dimensional (2D) approach to the assessment of emotion (the valence-arousal plane) with frontal alpha power asymmetry EEG (as a proposed index of valence) validated our visual music as an emotional stimulus. In Experiment 2, we used our synthetic stimuli to investigate possible underlying mechanisms of affective evaluation mechanisms in relation to audio and visual integration conditions between modalities (namely congruent, complementation, or incongruent combinations). In this experiment, we found that, when arousal information between auditory and visual modalities was contradictory [for example, active (+) on the audio channel but passive (−) on the video channel], the perceived emotion of cross-modal perception (visual music) followed the channel conveying the stronger arousal. Moreover, we found that an *enhancement effect* (heightened and compacted in subjects' emotional responses) in the aesthetic perception of visual music might occur when the two channels contained contradictory arousal information and positive congruency in valence and texture/control. To the best of our knowledge, this work is the first to propose a literature-based directive production of emotional visual music prototypes and the validations thereof for the study of cross-modally evoked aesthetic experiences in human subjects.

## Introduction

Previous psychological studies have revealed that movies are effective emotion inducers (Gross and Levenson, 1995; Rottenberg et al., 2007), and several studies have used films as emotional stimuli to investigate the biological substrates of affective styles (Wheeler et al., 1993; Krause et al., 2000). In addition, modern neuroimaging and neurophysiological techniques commonly use representational pictures (such as attractive foods, smiling faces, landscapes, and so on) from the International Affective Picture System (IAPS) as visual sources, and excerpts of classical music as auditory sources (Baumgartner et al., 2006a,b, 2007). Several studies have examined the combined influence of pictures and music but, to our knowledge, researchers have not used specifically composed, emotion-targeting cross-media content to elicit human emotion. It has been proposed that composing syncretistic artwork involves more complex types of practice by exploiting the *added values*, specific audio-visual responses that arise due to the synthesis of sounds and images, that occur as the result of naturally explicit A/V cues (Chion, 1994; Grierson, 2005). Hence, the practice of visual music, which composes abstract animations that simulate the *aesthetic purity of music* (Brougher et al., 2005), is naturally intermedia and has a synergy with modern digital auditory-visual (A/V) media. Thus, the affective value of visual music can embody important interactions between perceptual and cognitive aspects of the dual channels. Therefore, using abstract visual music to study emotion remains a promising but uninvestigated tool in human psychology.

**Figure 1:**
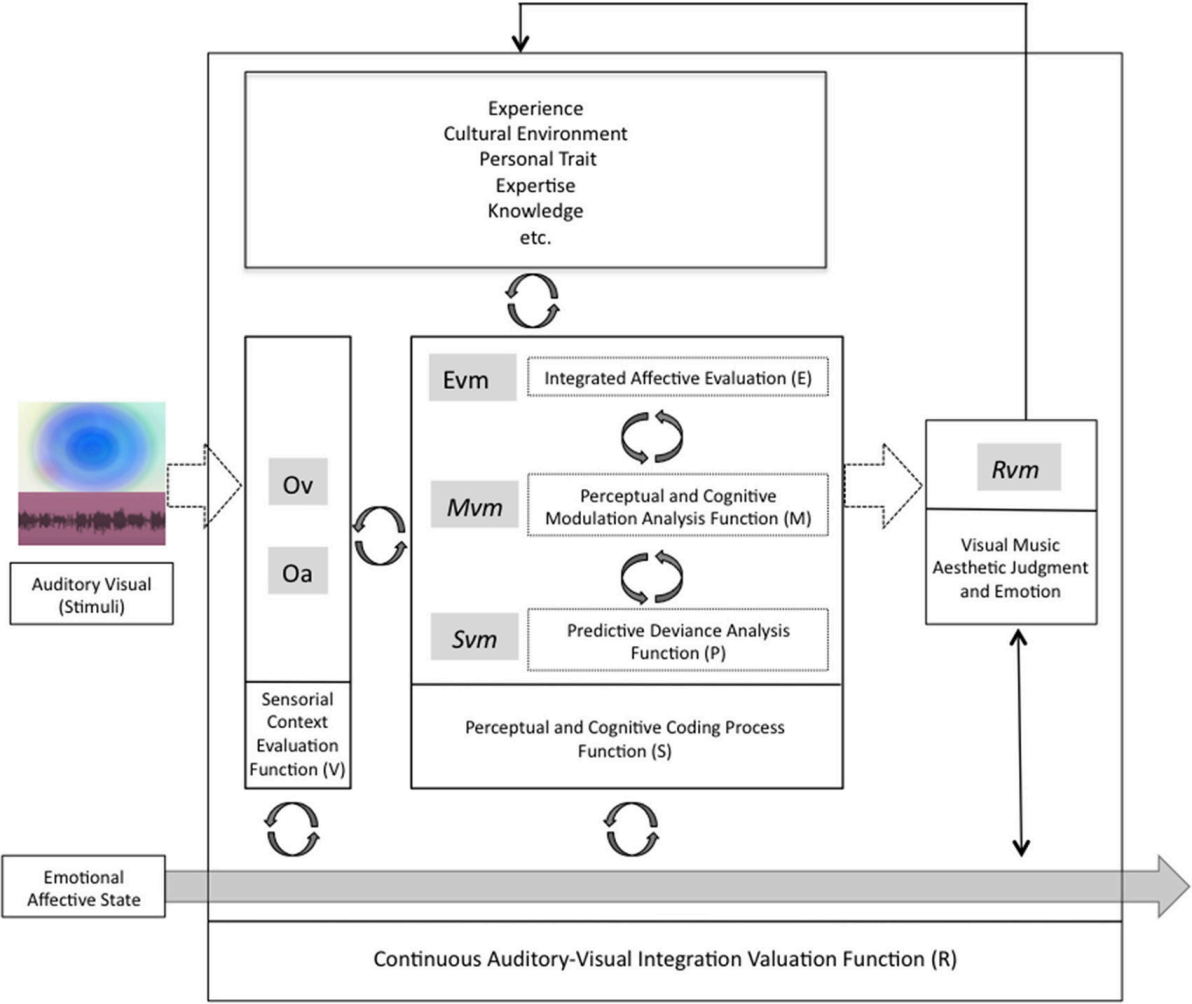
**A model of continuous auditory-visual modulation integration perception**. The diagram begins with the auditory-visual stimuli that are presented to the individual organisms and processed by evaluation functions (V) into their psychological values of (Ov) and (Oa). These psychological stimuli combine with continuous internal integration functions (S) through the modulation values (Mvm) and predictive deviation values (Pvm) into tentative implicit synthesizing responses (Evm). And the multisensory aesthetic evaluation function (R) transports them to elicit the immanent, holistic aesthetic emotion of the visual music (Rvm).

To understand the underlying mechanisms of the audio-visual aesthetic experience, we focused on the assessment of intrinsic structural and contextual aspects of stimuli, and the perceived aesthetic evaluations to formalize the process whereby visual music can suggest target emotions. Although we consider it premature to discuss the mechanism of the entire aesthetic experience within our study, we proposed a model of Continuous Auditory-Visual Modulation Integration Perception (Figure 1) to aid in the explanation of our research question. Our model is derived from the *Information Integration Theory* (Anderson, 1981), the *Functional Measurement Diagram* (Somsaman, 2004), and the *Information-Processing Model of Aesthetic Experience* (Leder et al., 2004), and describes how A/V integration might affect the overall aesthetic experience of visual music (Figure 1). Briefly explaining the three theories, the essential hypothesis of information integration theory requires three functions when a task involves certain responses from multiple stimuli: valuation (represented psychological values of multiple stimuli), integration (being combined into single psychological values), and response (being converted into an observable response) functions. The functional measurement diagram describes the perception of emotions in multimedia in the context of audio and visual information in relation to the information integration theory. And, the information-processing model of aesthetic experience describes how aesthetic experiences are involved with cognitive and affective processing and the formation of aesthetic judgments in a number of stages.

To investigate the perception of emotion via visual music stimuli, we started by examining the association of information-integration conditions (conformance, complementation, and contest) between two modalities (audio and video) and aesthetic perception (for details about integration conditions, see Cook, 1998, p. 98–106; Somsaman, 2004, p. 42–43). It is known that music/sound alters the meaning of particular aspects of a movie/visual stimulus (Marshall and Cohen, 1988; Repp and Penel, 2002), and that the ability of music to focus attention on a visual object stems from structural and semantic congruencies (Boltz et al., 1991; Bolivar et al., 1994). As researchers have recently emphasized that the semantic congruency between pairs of auditory and visual stimuli to enhance behavioral performance (Laurienti et al., 2004; Taylor et al., 2006), we hypothesized that the enhancement effect of visual music, by way of added value, might rely on the *congruency* of emotional information between unimodal channels (such as comparable valence and arousal information between auditory and visual channels). Therefore, our null hypothesis anticipates to see an enhancement effect of behavioral responses on the perception of emotion in the conformance combination condition of visual music due to the added value from the congruent emotional information provided by cross-modality. Accordingly, such a hypothesis might provide an indication of possible cases in which added values result in an enhancement effect via a cross-modal modulation evaluation process of the dual unimodal channel interplays as a *functional-information-integration* apparatus.

Our aim in this study, as a principled research paradigm, is a careful investigation of the aesthetic experience of auditory-visual stimuli in relation to the dual-channel combination conditions and the enhancement effect approaches from auditory-visual integrations by using emotional visual music prototypes. To examine our hypothesis, we conducted two different experiments, namely:

1. The creation of emotional visual music stimuli and the assessment of perceived emotional meanings of the stimuli according to modalities, and
2. The investigation of our hypothesis regarding the enhancement effect in relation to the integration conditions.

For Experiment 1, we first designed three positive emotional visual music stimuli by producing audio and video content in accordance with literature-based formal property directions to evoke target-emotions. We then presented our compositions as unimodal (audio only or visual only) or cross-modal (visual music) stimuli to subject groups and surveyed psychometric affective responses (self-rating of emotion), from which three indices were derived (evaluation, activity, and potency, which are equivalent to valence, arousal, and texture/control, respectively). In this experiment, we focused on a two-dimensional (2D) representation (valence and arousal) to validate the affective meaning of our visual music in accordance with the *circumplex model of affect* (Posner et al., 2005). Finally, we examined electroencephalography (EEG) responses as physiological internal representations of valence (in other words, as represented by frontal alpha asymmetry) to the representation of visual music as a partial emotional validation of visual music. In our main experiment (Experiment 2), we investigated the auditory-visual aesthetic experience, with a particular focus on added-value effects that result in affective enhancement as a functional-information-integration apparatus. To investigate this, we included two additional visual music compositions (negative in valence) created by a solo media artist to our visual music stimuli. We separated the unimodal stimuli from the five original visual music stimuli into independent audio-only and visual-only channel information (named A1–A5 and V1–V5, respectively), and assessed the affective emotion thereof via our subjects' self-ratings (as in Experiment 1). Finally, we cross-matched the unimodal information of altered visual music, forming three combination conditions (conformance, complementation, and contest) of multimedia combinations (Figure S1), and compared the aesthetic affective responses of viewers (self-rated) to investigate our enhancement effect hypothesis using the nine visual music stimuli (five original visual music stimuli and four altered visual music stimuli).

## Experiment 1: emotional visual music design and validation

In this experiment, we explained the scheme for the construction of each modality of the three visual music stimuli, and we assessed the subjects' perceptions of our target emotion via a 2D representation (valence vs. arousal using indices constructed from the self-ratings). We also assessed the electrophysiological response of the participants relative to valence perception (positive vs. negative) and EEG frontal alpha asymmetry to validate perceived and potential emotion elicitation. The aim of this first experiment was to validate the construction paradigm and the emotion-targeting properties of our abstract visual music movies.

### Methods

#### Participants

For the preliminary experiment, we recruited 16 people from the Graduate School of Culture Technology (GSCT) in the Korean Advanced Institute of Science and Technology (KAIST) and from Chungnam University, Daejeon, South Korea. Advertisements to recruit aesthetic questionnaire participants for two different groups were posted on the bulletin boards to collect native Korean-language speakers with no special training or expertise in the visual arts or in music (people who could not play any musical instruments or who only played instruments as a hobby and had < 3 years' of lessons). The two groups were the unimodal behavioral survey group and the cross-modal survey with EEG recording group. The unimodal survey group subjects [mean age = 26.12, sex (m:f) = 4:4, minimum age = 24, maximum age = 29] attended individual survey sessions at an allocated time. Each subject attended the experimental session for about 20 min, and had the option to enter a draw to win an on-line shopping voucher worth $20 in return for their participation. All participants completed a questionnaire detailing their college major, age, gender, and musical or visual background. The participants had various kinds of degrees, such as broadcasting, computational engineering, bio-neuro science, and business management, while no subject was a musician or an art major. The cross-modal survey group subjects [mean age = 22.5, sex (m:f) = 4:4, minimum age = 20, maximum age = 26] attended the experimental session in the morning and spent about 60 min including preparation, recording the EEG and the behavioral survey session, and after experiment debriefing. The subjects were given $30 per hour as compensation. All participants were certified as not suffering from any mental disorder, not having a history of drug abuse of any sort, and provided a signed consent form after receiving an explanation about the purpose of and procedure for the experiment. In our cross-modal group, no subject was a music or an art major. The study was approved by the Institutional Review Board of KAIST.

#### Stimuli composition

The notion of the existence of some universal criteria for beauty (Etcoff, 2011), together with the consideration of *formalist* (aesthetic experience relies on the intrinsic sensual/perceptual beauty of art) and *contextual* (aesthetic experience depends on the intention/concept of the artist and the circumstances of display) theories (for reviews, see Shimamura, 2012; Redies, 2015) makes it possible to create the intrinsic positive valence of auditory-visual stimuli based on an automatic evaluation of esthetic qualities. Hence, we constructed abstract animations synchronized with music that could convey jointly equivalent positive emotional connotations to assess positive emotion-inducing visual music. In comparison to existing visual music, the new stimuli with a directive design could be advantageous for conducting emotion-inducing empirical research because they allow for the inspection of structural and contextual components, and provide information about production to a certain extent. They can also balance production quality differences among stimuli more easily than when using existing artworks. Furthermore, they remove the familiarity effect that seems to be a critical factor in the listener's emotional engagement with music (Pereira et al., 2011).

To conceptualize the target emotion and to discriminate among the perceived affective responses to the stimuli, we took a dimensional approach, which identifies emotions based on the placement on a small number of dimensions and allows a dimensional structure to be derived from the response data (for a review of the prominent approaches to conceptualizing emotion, see Sloboda and Juslin, 2001, p. 76–81). We chose and characterized three “positive target-emotions” (happy, relaxed, and vigorous) that can be distinctly differentiated from each other when placed on a 2D plane similar to the circumplex model (Posner et al., 2005). Please see Figure S2.

- Happy: high-positive valence and neutral-active arousal.
- Relaxed: mid-positive valence and mid-passive arousal.
- Vigorous: neutral-positive valence and high-active arousal.

We then focused on previously published empirical studies that examined the iconic relationships between the separate structural properties of visual stimuli and emotion (Takahashi, 1995; Somsaman, 2004) and auditory stimuli and emotion (Gabrielsson and Lindstrom, 2001; Sloboda and Juslin, 2001) to customize our directive production guidelines for our team of creative artists. By considering constructivist theory (Mandler, 1975, 1984), we assembled important structural components that matched the targeted emotional expressions via a broad but non-exhaustive review of previous research on the correlation of emotional elicitation in auditory (Table 1) and visual (Table 2) cues. Based on the idea that not only the formal properties of stimuli but also the intention of the artist and the circumstances can affect aesthetic experience to a large extent (see Redies, 2015), we further briefed our creative artists about the use of stimuli in the experiment. The creative artists cooperated fully to create visual music content conveying targeted emotions to the viewers by complying with the directive guidelines to output them as positive affection-inducing stimuli.

**Table 1:** **Summary of musical design structural guidelines selected from reviewed studies and artists' decisions**.

**Structural component**	**Happy**	**Relaxed**	**Vigorous**	**Studies linked to the correlation of structural components and emotion**
Harmony	Consonant	Consonant	Consonant (with harmonic tensions such as b9, 9, 11, and 13, for example)^*^	For comprehensive reviews for studies of correlations between musical expressions and emotions, see (Bunt and Pavlicevic, 2001; Gabrielsson and Lindstrom, 2001).
Tonal Modality	Major (in F^#^)^*^	minor (in C)^*^ moving to Major (E^b^)^*^Alternation	minor (in D)^*^	
Melodic intervals	Wide Range, High-Pitched	Narrow Range, Low-Pitched	Complex (narrow to wide) Range, Large Intervals	
Pitch level	High	Mid	High+Low	
Loudness and loudness variation	Small few/no change	Soft few/no change	Loud rapid changes	
Articulation	Staccato	Legato	Complex (staccato + legato)	
Tempo and pulse	Firm pulse, fast and lively (112 bpm, Dotted Tempo, 4/4 meter)^*^	Relaxed pulse, gentle and slow pulse (around 60 bpm, 4/4 meter)^*^	Firm pulse, variedly quick and rapid (from 174 to 87 bpm, 4/4 meter)^*^	
Adjectives for timbre and texture (depicted by main instruments and musical style)	Bright and playful (female vocal, latin percussive)^*^	Calm, serene, with slow breathing (korean daegum flute, ethnic)^*^	Tension and alleviation (electronic synthesizer lead, drum and bass)^*^	
Pitch contour and melodic direction	Slightly Ascending at the Ending	Descending toward Ending	Building Up & Down	
	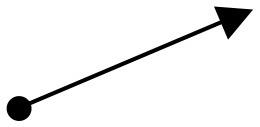	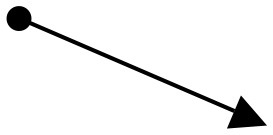	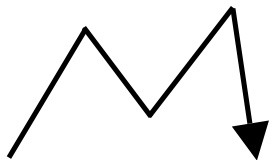	
Motive phrase melodic motion	Intervallic leaps, descending pitch contour	Stepwise + Leaps, ascending pitch contour	From stepwise to intervallic leaps, high complexity	

*Prototypical directive guidelines provided information about basic settings in important musical structural expressions to generate the associated target emotional meaning in the music*.

* *Sub-element of the component chosen by the artist considering the target emotion of the music production*.

**Table 2:** **Summary of visual design structural guidelines selected from reviewed studies and artists' decisions**.

**Structural component**	**Happy**	**Relaxed**	**Vigorous**	Studies of related variables of visual components^**^
**Thematic milieus (adjective)**	Warm, delight, cheerful, joyous	Calm, soothing, serene, tranquil	Exploding, lively, sprightly, exciting	Lundholm, 1921; Hevner, 1935
**Main motive object shape**	Water droplet: circular form 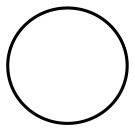 (Spherical)	Water waves: S-curve flows 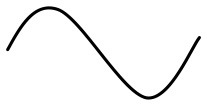 Then (Kaleidoscopic, Oval, Rhombus, Hexagon)^*^	Water Vortex: 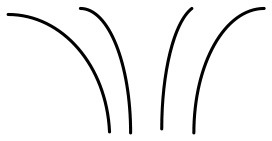 up heaving (Symmetrical Pattern, Petaloid)^*^	Hevner, 1935; Lyman, 1979; Takahashi, 1995
Main object movement (secondary movement)^*^	Repetition of circles	Descending S-curves then focusing in^*^	Expanding out then repetition of rising bursts^*^	Poffenberger and Barrows, 1924; Takahashi, 1995
Main color pallets (secondary colors)^*^	Red, orange, yellow 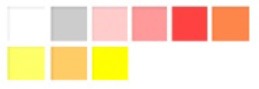 (Green, Blue)^*^	Blue, green, purple, white, brown 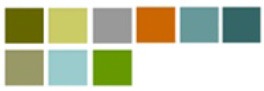	Blue, green, yellow, white 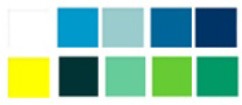 (Pink)^*^	Hevner, 1935; Wexner, 1954; Lyman, 1979
**Object kinetics and moving vectors**	Uphill Slant (Lower Left to Upper Right), 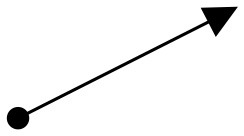 Upper Right to Lower Left 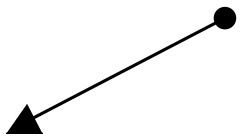	Horizontal (Left To Right), 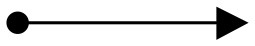 Downhill Slant (Upper Left to Lower Right) 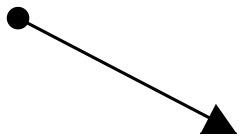	Vertical Upward, 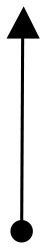 Expansion, Fast Cuts, Contrasts	Block, 2013; Zettl, 2013
**Editorial scene rhythm**	Medium	Slow	Quick	Block, 2013; Zettl, 2013
**Sequence transition**	Strong and fast changes, jump cuts, long exposure of objects	Slow and weak changes, smooth and dissolve, small number of scenes	Contrasting between different images, jump cuts, many scene changes	Block, 2013; Zettl, 2013

*Prototypical directive guidelines provided information about basic settings in important visual and animation structural expressions to generate the associated target emotional meaning in the abstract animation*.

* *Sub-element of the component chosen by the artist considering the target emotion of the music production*.

** *Selective studies vary in methodological approaches to investigating the influence of the related visual component and its expressions regarding emotion*.

##### Audio-only stimuli production

Three 60-s, non-lyrical (containing no semantic words), and consonant music pieces were created by a music producer based on the directive design guidelines (Table 1). Basic literature-based information for musical structure properties, such as harmony, modality, melody, metrical articulation, intensity, rhythm, instrument, and tempo, were suggested (for reviews, see Bunt and Pavlicevic, 2001; Gabrielsson and Lindstrom, 2001). However, the artist appointed to create emotion-inducing music noted particular details regarding the decisions. For example, the research team suggested modality directions based on reviewed studies (such as “Major” for “happy”), and the artist chose the tonic center of the key (F#), which can be a basic sub-element of the modality. All the music was created in a digital audio workstation that was equipped with hardware sound modules, such as a Kurzweil K2000, Roland JV-2080, and Korg TR-Rack, and music recording/sequencing programs, such as Digidesign's Pro Tools, Motu's Digital Performer, and Propellerhead's Reason, as well as other audio digital signal processing (DSP) plug-ins, such as Waves Gold Bundle. The final outputs were exported as digitized sound files (44.1 k sampling rate, 16-bit stereo).

##### Video-only stimuli production

Three colored, full-motion, and abstract animations that included forms (shapes), movement directions, rhythm, colors, thematic milieus, scene-changing velocities, and animation characteristic directions were created based on the guidelines (Table 2). The collaborative team consisted of two visual artists, one of whom designed the image layout and illustration with shapes and colors, while the other created the animated motion graphics for it. As with the audio stimuli, we suggested directive guidelines for the overall important structural factors to the artists, and they noted detailed sub-elements. To create the animations, the artists used a digital workstation equipped with Adobe Photoshop, Premier, Max/MSP with Jitter, After Effects, and a Sony DCR-HC1000. The final visual stimuli consisted of QuickTime movie (.mov) files that were encoded using Sorenson Video 3 (400 × 300, NTSC, 29.97 non-drop fps).

##### Visual music integration

**Figure 2:**
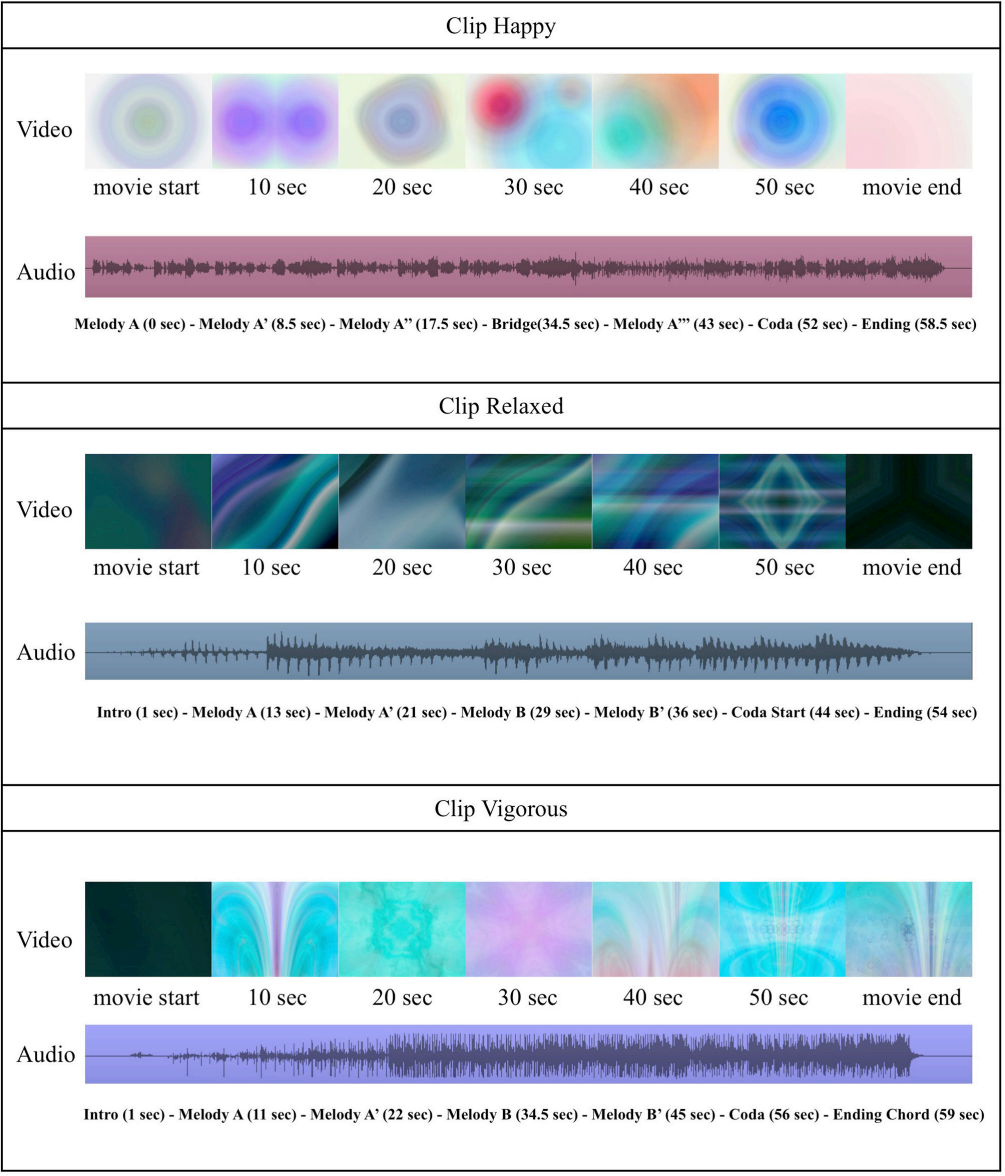
**Snapshot information about the visual channel, the auditory channel, and structural variations in the temporal streams of our three positive emotion visual music stimuli**.

For each visual music integration, the motion graphic artist arranged the synchronization of visual animations to its comparable music (for example, happy visual animation synchronized to happy music) while taking the directive motion guidelines (Table 2) into account. In other words, the movements of the visual animation components, sequence changes, kinetics, and scene-change speed evolved over time in accordance with the directive guidelines while incorporating a good accompaniment to the compatible formal and contextual changes in the music (such as accents in rhythms, climax/high points, the start of a new section, and so on). An illustrative overview of the three visual music stimuli is provided in Figure 2, and the content is available online at https://vimeo.com/user32830202.

#### Procedure

Prior to each experiment, regardless of the modalities, all participants were fully informed that they were participating in a survey for a study investigating aesthetic perception for a scientific research project in both written and verbal forms. We distributed a survey kit, which included information about the purpose of the survey, questions to be answered by the participants regarding demographic information, major subjects, and music/art educational backgrounds (if they played any instruments, how long they had experienced musical/visual art training), and an affective questionnaire form for each presentation. Our affective questionnaire consisted of 13 pairs of scales that were generated by referencing a previous study (Brauchli et al., 1995) that used nine-point scale ratings for bipolar sensorial adjectives to categorize the emotional meanings of the perceived temporal affective stimuli (Figure S3). We also explained verbally to our participants how to respond to the emotion-rating tasks on the bipolar scales. The consent of all subjects was obtained before taking part in the study, and the data were analyzed anonymously. All our survey studies, irrespective of modal conditions, were exempt from the provisions of the Common Rule because any disclosure of identifiable information outside of the research setting would not place the subjects at risk of criminal or civil liability or be damaging to the subjects' financial standing, employability, or reputation.

##### Unimodal presentation

For the unimodal group, we conducted 20-min experimental sessions with each individual participant in the morning (10–11 a.m.), afternoon (4–5 p.m.), or evening (10–11 p.m.) time slots, depending on the subject's availability. A session consisted of a rest period (5 s) followed by the stimuli presentation (60 s), repeated in sequence for all stimuli. Three audio-only and three visual-only stimuli were shown to subjects via a 19-inch LCD monitor (Hewlett Packard LE1911) with a set of headphones (Sony MDR-7506). The stimuli presentation was in pseudo-random order by altering the play order of modality blocks (audio only or video only) and by varying individual stimuli sequences within each block to obtain the emotional responses of subjects for all unimodal stimuli while avoiding an unnecessary modality difference effect; for example, V1-V3-V2-A1-A2-A3 for subject 1, A2-A1-A3-V2-V1-V3 for subject 2, V3-V2-V1-A3-A2-A1 for subject 3, and so on. The audio-only stimuli accompanied simple black screens, and the video-only stimuli had no sound on the audio channels. Each subject completed the questionnaire while watching or listening to the stimulus (see Figure S4a). Subjects were not given a break until the completion of the final questionnaire in an effort to preserve the mood generated and to avoid unexpected mood changes caused by the external environment.

##### Cross-modal presentation and EEG recordings

The cross-modal tests were performed in the morning (9–12 a.m.) and, excluding the preparation and debriefing, the EEG recording and behavioral response survey procedures took ~15–20 min per subject. During each session, participants (*n* = 8) were seated in a comfortable chair with their heads positioned in custom-made head holders placed on a table in front of the chair (Hard PVC support with a soft cushion to reduce head movement and to maintain a normal head position facing the screen). Each participant was presented with the visual music, had their heads facing the computer screen (the head to screen distance was 60 cm from a 19-inch LCD monitor; Hewlett Packard LE1911), and was given a set of headphones (Sony MDR-7506). Three visual music stimuli were shown in pseudo-random order to avoid sequencing effects. Resting EEGs were recorded for 20 s as a baseline before each watching session to allow for a short break before the subjects moved on to the next stimulus, thus avoiding excessive stress and providing a baseline time calculation for the resting alpha frequency peak (see Section Data Analysis, EEG analysis). The subjects answered the survey relating to emotion after completing the EEG recording of watching the visual music stimulus (Figure S4b) to allow for complete focus on the presentation of the stimuli and to avoid unnecessary noise artifacts in the EEG recordings. EEG recordings were digitalized at a frequency of 1,000 Hz over 17 electrodes (Ag/AgCl, Neuroscan Compumedics) that were attached according to the 10–20 international system with reference and ground at left and right earlobes (impedance < 10 k ohms), respectively. Artifacts resulting from eye movements and blinking were eliminated based on the recording of eye movements and eye blinking (HEO, VEO), using the Independent Component Analysis (ICA) in the EEGLAB Toolbox®(Delorme and Makeig, 2004). Independent components with high amplitude, high kurtosis and spatial distribution in the frontal region (obtained through the weight topography of ICA components) were visually inspected and removed when identified as eye movement/blinking contaminations. Other muscle artifacts related to head movements were identified via temporal and posterior distribution of ICA weights, as well as via a high-frequency range (70–500 Hz). The EEG recordings were filtered using a zero-phase IIR Butterworth bandpass filter (1–35 Hz). All of the computer analyses and EEG processing were performed using MATLAB® (Mathworks, Inc.).

#### Data analysis

##### Emotion index and validity

Although the 13 pairs of bipolar ratings from the survey could provide useful information about the stimuli independently of each other, there was a need to divide them into smaller dimensions to identify emotions based on their position in a small number of dimensions. While the circumplex model is known to capture fundamental aspects of emotional responses, in order to avoid losing important aspects of the emotional process as a result of dividing them into too many dimensions, we indentified three indices of evaluation, activity, and potency. We referred to the *Semantic Differential Technique* (Osgood et al., 1957), and extracted the three indices by calculating mean values of the ratings of four pairs per index from the original 13 pairs used in our surveys. Specifically, the evaluation index assimilates “valence,” and its value was obtained from the mean ratings of the happy-sad, peaceful-irritated, comfortable-uncomfortable, and interested-bored scales. The activity factor represents “arousal,” and is the average of the tired-lively, relaxed-tense, dull-energetic, and exhausted-fresh scales. The potency factor reflects “control-related,” and was derived from the unsafe-safe, unbalanced-balanced, not confident-confident, and light-heavy scales. We did not include the calm-restless scale in the activity index because we found (after completion of the surveys) that there was a discrepancy in the Korean translation, which showed “unmatched” for the bipolarity pairing. The final indices (evaluation, activity, and potency) were rescaled from the nine-point scales to a range of [−1, 1], as shown in Figure S5.

##### EEG analysis

For the EEG, we adopted a narrow-band approach based on the Individual Alpha Frequency (IAF; Klimesch et al., 1998; Sammler et al., 2007) rather than a fixed-band approach (for example, a fixed alpha band of 8–13 Hz). This approach is known to reduce inter-subject variability by correcting the true range of a narrow-band based on an individual resting alpha frequency peak. In other words, prior to watching each clip (subjects resting for 20 s with their eyes focused on a cross [+]), the baseline IAF peak of each subject was calculated (clip 1: 10.7 ± 1.5 Hz; clip 2: 10.1 ± 0.8 Hz; clip 3: 10.1 ± 1.3 Hz). The spectral power of the EEG was calculated using a fast-Fourier transform (FFT) method for consecutive and non-overlapping epochs of 10 s (for each clip, independent baseline and clip presentation). In order to reduce inter-individual differences, the narrow band ranges were corrected using the IAF that was estimated prior to each clip according to the following formulas: Theta ([0.4–0.6] × IAF Hz), lowAlpha1 ([0.6–0.8] × IAF Hz), lowerAlpha2 ([0.8–1.0] × IAF Hz), upperAlpha band ([1.0–1.2] × IAF Hz) and Beta ([1.2–2] × IAF Hz). From the power spectral density, the total power in each band was calculated and then log transformed (10log_10_, results in dB) in order to reduce skewness (Schmidt and Trainor, 2001). The frontal alpha power asymmetry was used as a valence indicator (Coan and Allen, 2004), and was calculated for the first 10 seconds of recording (10log_10_) to monitor emotional responses during the early perception of the visual music (Bachorik et al., 2009) and the delayed activation of the EEGs (also associated with a delayed autonomic response; Sammler et al., 2007). For the overall topographic maps over time (considering all 17 channels; EEGLAB topoplot), the average power in each band divided by the average baseline power in the same band was plotted for each 10 s epoch from the baseline to the end of the presentation (subject-wise average of 10log_10_(P/P_base_), where P is the power and P_base_ is the baseline power in the band; see **Figure 4** and Figure S6). All statistical analyses were performed on the log-transformed average power estimates.

##### Statistical analysis

To inspect the perceived emotional meanings of each unimodal presentation, we compared the means of the evaluation, activity, and potency indices (three factors of “affection” as dependent variables) across the audio only and across the video only. We performed a multivariate analysis of variance (two-way repeated measure MANOVA) using two categories as between subject factors (independent variables), “modality” (two levels: audio only and visual only) and “target-emotion” (three levels: happy, relaxed, and vigorous). This analysis examines three different null hypotheses:

1. “Modality” will have no significant effect on “emotional assessment” (evaluation, activity, and potency),
2. “Target-emotion” will have no significant effect on “emotional assessment,” and
3. The interaction of “modality” and “emotion” will have no significant effect on “emotional assessment.”

We then performed follow-up one-way repeated measure analysis of variance (one-way repeated measure ANOVA) tests for the three indices combined with the same within-subject factor “target-emotion” for each modality. We checked typical assumptions of a MANOVA, such as normality, equality of variance, multivariate outliers, linearity, multicollinearity, and equality of covariance matrices and, unless stated otherwise, the test results met the required assumptions. ANOVAs that did not comply with the sphericity test (Mauchly's test of sphericity) were reported using the Hyunh-Feldt correction. *Post-hoc* multiple comparisons for the repeated measure factor “clip” were performed using a paired *t*-test with a Bonferroni correction.

To inspect the perceived emotional meanings of the visual music stimuli, we conducted a one-way ANOVA to examine the means of evaluation, activity, and potency indices (three factors of “affection” as dependent variables) for each “target-emotion” clip. Considering the mixed subject groups (identical subjects in audio only and video only, but different subjects in the visual music group), we then conducted non-parametric Kruskal-Wallis *H*-tests (K–W test) to see if “modality” would have an effect on “emotional assessment” (evaluation, activity, and potency) in the same “target-emotion” group (“happy” target-emotion stimuli: A1, V1, and A1V1), and to examine whether the interaction of “modality” and “emotion” would have an effect on “emotional assessment.”

For the frontal asymmetry study of the EEGs, we used a symmetrical electrode pair, F3 and F4, for upperAlpha band powers (see EEG Analysis Section for a definition of upperAlpha band from IAF estimation) to calculate lateral-asymmetry indices by power subtraction (power of F4 upperAlpha—power of F3 upperAlpha). We performed a Pearson's correlation between the valence index (evaluation) and the frontal upperAlpha asymmetry in order to validate our index representation of valence through electrophysiology. For each clip, we performed a paired *t*-test between F4 and F3 upperAlpha values to quantify the frontal alpha asymmetry.

All statistical tests were performed using the Statistical Package for Social Science (SPSS) version 23.0.

### Results

#### Unimodal stimuli

##### Audio only

**Figure 3:**
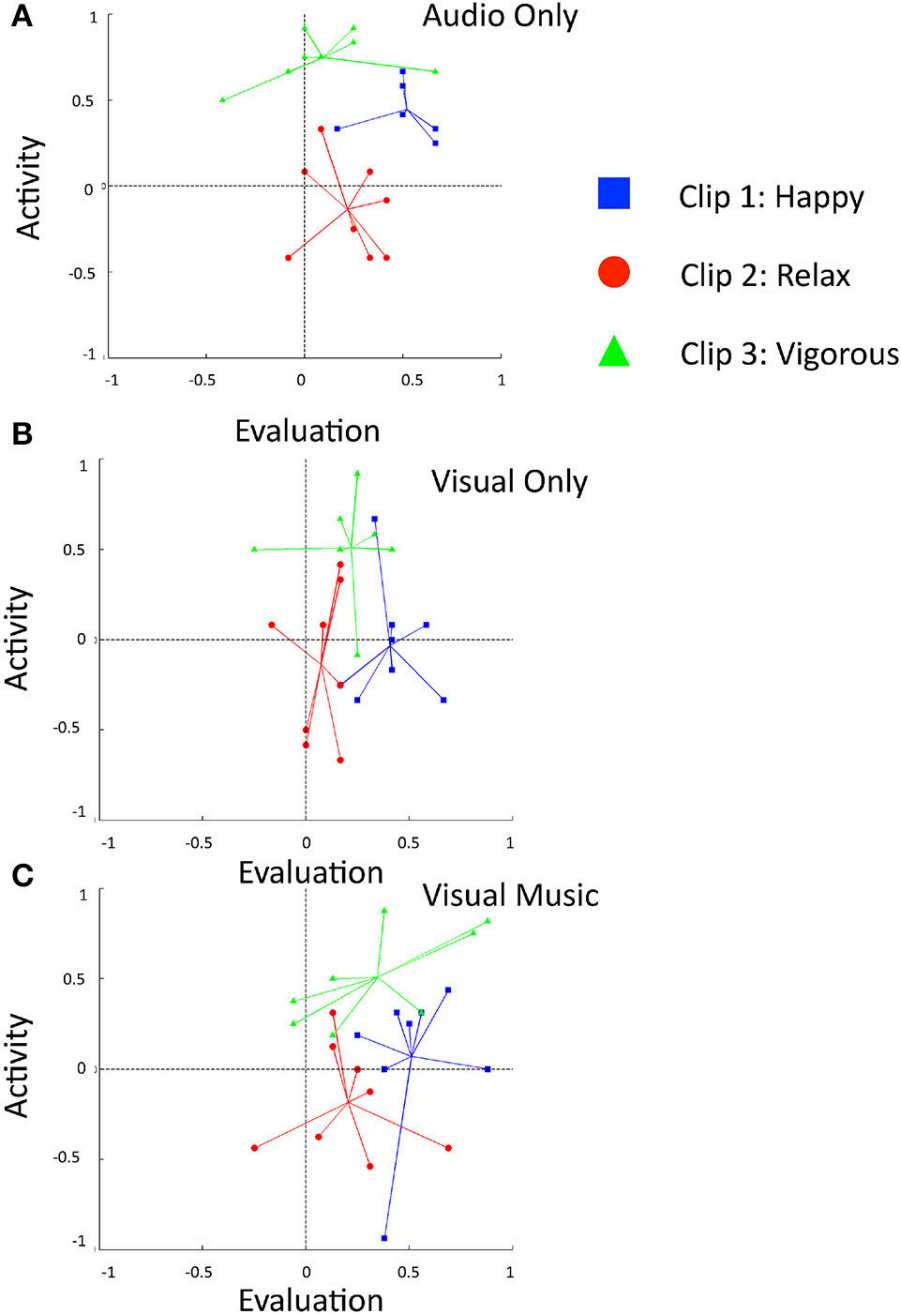
**Comparison of connotative meanings of three clip stimuli using the evaluation and activity indices into 2D planes**. Subjects' responses are shown for the conditions **(A)** audio only, **(B)** visual only, and **(C)** visual music (combined auditory and visual stimuli).

We obtained the affective characteristic ratings of each auditory-only stimulus, as shown in Table 3 and Figure 3. A one-way ANOVA was conducted to compare the effect of the presentations on the three indices. The result showed that the effect of the clips on the levels of all three indices was significant; evaluation [*F*_(2, 21)_ = 7.360, *p* = 0.004, η^2^ = 0.412], activity [*F*_(2, 21)_ = 39.170, *p* = 0.000, η^2^ = 0.789], and potency [*F*_(2, 21)_ = 14.948, *p* = 0.000, η^2^ = 0.571]. We found that clip 1 received a significantly higher rating in the evaluation index than did clip 3 (*p* = 0.003). The clip 2 received a significantly lower rating in the activity index than did clip 1 (*p* = 0.000), and clip 3 (*p* = 0.000). Clip 3 received a significantly higher rating in the activity index (*p* = 0.045) and a lower rating in the potency index (*p* = 0.001) than did clip 1, and a significantly higher rating in the activity index (*p* = 0.000) and lower indexing in the potency index (*p* = 0.000) than did clip 2. The results indicate that the valence level (evaluation value) of all three auditory stimuli was perceived as positive, with the clip 1 “happy” showing the highest positive level of valence. The variations in the activity index showed “relaxed” as the lowest (mean = −0.06, neutral-passive) and “vigorous” as the highest (mean = 0.81, high-active) arousal levels, respectively.

**Table 3:** **Mean and standard deviation comparison for evaluation, activity, and potency indices of all three stimuli in three different modalities**.

**Stimuli**	***N***	**Evaluation**		**Activity**		**Potency**	
		**Mean**	***SD***	**Mean**	***SD***	**Mean**	***SD***
Audio Only Clip 1 (A1)	8	0.60	0.17	0.54	0.16	0.27	0.15
Visual Only Clip 1 (V1)	8	0.47	0.16	0.06	0.33	0.08	0.36
A1V1 (A1V1)	8	0.51	0.20	0.07	0.43	0.34	0.17
Audio Only Clip 2 (A2)	8	0.32	0.18	−0.06	0.28	0.32	0.29
Visual Only Clip 2 (V2)	8	0.15	0.11	−0.02	0.42	0.08	0.36
A2V2 (A2V2)	8	0.13	0.27	−0.27	0.24	−0.11	0.24
Audio Only Clip 3 (A3)	8	0.18	0.30	0.81	0.12	−0.28	0.27
Visual Only Clip 3 (V3)	8	0.28	0.25	0.59	0.27	0.26	0.22
A3V3 (A3V3)	8	0.34	0.37	0.51	0.27	0.13	0.35

##### Video only

For the visual-only content, we obtained the mean values as shown in Table 3 and Figure 3. A One-way ANOVA test showed that the effect of the clips on the levels of the three indices was significant in the evaluation [*F*_(2, 21)_ = 6.262, *p* = 0.007, η^2^ = 0.373] and activity indices [*F*_(2, 21)_ = 7.488, *p* = 0.004, η^2^ = 0.416]. The evaluation index of clip 1 obtained a significantly higher rating than did clip 2 (*p* = 0.006). The activity index showed clip 3 received a significantly highest rating than did clip 1 (*p* = 0.017) and clip 2 (*p* = 0.005). No significantly different effect of the clips was reported for the potency index. The results indicate that all visual-only animations were perceived positively at valence level; however, there were no clear distinctions of the perceived emotional information (evaluation, activity, and potency) among the three animations (V1~V3), as was partially shown in the audio clips (A1~A3). The animation “vigorous” showed the highest activity (mean = 0.59, high-active) rating, while both the animations “happy” and “relaxed” showed activity ratings that indicated low-level arousal experienced by the subjects for these two animations (mean = 0.06, neutral-passive and mean = −0.02, neutral-passive, respectively).

We conducted two-way repeated measure MANOVA tests with the three indices and two between-subject factors (“modality” and “target-emotion”), and Mauchly's test indicated that the assumption of sphericity had been violated, $\chi_{\left(2\right)}^{2}$ = 14.244, *p* = 0.001; therefore, degrees of freedom were corrected using Huynh-Feldt estimates of sphericity (ε = 0.77), and the results are shown in Table 4. The results of the 2 × 3 MANOVA with modality (audio only, visual only) and target-emotion (happy, relaxed, vigorous) as between-subject factors show a main effect of emotion, [*F*_(3.57, 74.98)_ = 17.80, *p* = 0.000, $\eta_{p}^{2}$ = 0.459], and an interaction between modality and emotion [*F*_(3.57, 74.98)_ = 3.548, *p* = 0.013, $\eta_{p}^{2}$ = 0.145]. The results indicate that “target-emotion” and the interaction of “modality and target-emotion” have significantly different effects on evaluation, activity, and potency levels.

**Table 4:** **Results of Two-Way Repeated Measure MANOVA using the Huynh-Feldt Correction following a violation in the assumption of sphericity**.

**Effect**	**Type III sum of squares**	***df***	**Mean square**	***F***	**Sig**.	**Partial eta squared**
AestheticEmotion	1.527	1.785	0.855	11.018	0.000	0.208
AestheticEmotion ^*^ Modality	0.324	1.785	0.182	2.340	0.109	0.053
AestheticEmotion ^*^ Emotion	4.933	3.570	1.382	17.796	0.000	0.459
AestheticEmotion ^*^ Modality ^*^ Emotion	0.984	3.570	0.257	3.548	0.013	0.145
Error(Affection)	5.821	74.978	0.078			

*The analysis checks for effects of variables in modality and target emotion categories on affection information (evaluation, activity, and potency)*.

#### Cross-modal stimuli

##### Behavioral response

The ratings for the cross-modal subjects were obtained, and were distributed as shown in Table 5 and Figure 3. The simple placement of evaluation and activity values on the 2D plane illustrates the characteristics of our stimuli at a glance, and we can see that they indicate positive in emotional valence (mean evaluation index ≥ 0), irrespective of the modality of the presentation (Figure 3). Clip 1, “happy,” targeted a high-positive valence and neutral-active arousal emotion, and indicated the comparable perception of the subjects (evaluation: 0.51 ± 0.20; activity: 0.07 ± 0.43); clip, 2 “relaxed,” a mid-positive valence and mid-passive arousal, displayed a tendency similar to the viewers' responses (evaluation: 0.13 ± 0.32; activity: −0.27 ± 0.24), while clip 3, “vigorous,” showed a neutral-positive valence and high-active arousal (evaluation: 0.34 ± 0.37; activity: 0.51 ± 0.27).

**Table 5:** **Results of the Kruskal-Wallis Test comparing the effect of our designed emotional stimuli on the three perceived emotion assessment indices (evaluation, activity, and potency)**.

	**Emotion assessment indices**	***N***	**Mean**	**Std. Deviation**	**Min**	**Max**	**Percentiles**		
							**25th**	**50th (Median)**	**75th**
**DESCRIPTIVE STATISTICS (NPar Test)**									
Clip 1 Target-Emotion ‘Happy’	Evaluation	24	0.526	0.178	0.250	0.880	0.375	0.531	0.688
	Activity	24	0.224	0.387	−0.940	0.750	0.000	0.281	0.438
	Potency	24	0.237	0.204	−0.060	0.630	0.063	0.250	0.359
	Total	24	24.330	26.811	1.000	61.000	1.000	11.000	61.000
Clip 2 Target-Emotion ‘Relaxed’	Evaluation	24	0.198	0.227	−0.310	0.690	0.063	0.219	0.359
	Activity	24	−0.117	0.328	−0.560	0.560	−0.422	−0.188	0.125
	Potency	24	0.086	0.344	−0.500	0.690	−0.172	−0.031	0.359
	Total	24	28.670	25.481	2.000	62.000	2.000	22.000	62.000
Clip 3 Target-Emotion ‘Vigorous’	Evaluation	16	0.268	0.306	−0.310	0.880	0.063	0.281	0.469
	Activity	16	0.635	0.256	0.000	0.940	0.500	0.719	0.813
	Potency	16	0.000	0.339	−0.750	0.690	−0.250	−0.031	0.250
	Total	16	33.000	25.022	3.000	63.000	3.000	33.000	63.000
	**Emotion assessment indices**		**Clip ID**	***N***	**Mean Rank**	**Chi-Square**	***df***	**Asymp. Sig**.	**Effect Size (**η^2^**)**
**RANKS (KRUSKAL-WALLIS TEST)**									
Clip 1 Target-Emotion ‘Happy’	Evaluation	A1	8	**16.060**	3.227	2	0.199	0.140	
		A1V1	8	11.380					
		V1	8	10.060					
		Total	24						
	Activity^*^	A1	8	**19.250**	11.409	2	0.003	0.496	
		A1V1	8	10.130					
		V1	8	8.130					
		Total	24						
	Potency^*^	A1	8	14.060	6.244	2	0.044^*^	0.271	
		A1V1	8	**15.880**					
		V1	8	7.560					
		Total	24						
Clip 2 Target-Emotion ‘Relaxed’	Evaluation	A2	8	**16.380**	3.674	2	0.159	0.160	
		A2V2	8	10.560					
		V2	8	10.560					
		Total	24						
	Activity	A2	8	**14.250**	2.379	2	0.304	0.103	
		A2V2	8	9.380					
		V2	8	13.880					
		Total	24						
	Potency^*^	A2	8	**17.310**	6.687	2	0.035	0.291	
		A2V2	8	8.250					
		V2	8	11.940					
		Total	24						
Clip 3 Target-Emotion ‘Vigorous’	Evaluation	A3	8	10.060	1.441	2	0.487	0.063	
		A3V3	8	13.630					
		V3	8	**13.810**					
		Total	24						
	Activity^*^	A3	8	**17.440**	6.143	2	0.046	0.267	
		A3V3	8	9.190					
		V3	8	10.880					
		Total	24						
	Potency^*^	A3	8	6.560	8.551	2	0.014	0.372	
		A3V3	8	15.130					
		V3	8	**15.810**					
		Total	24						

*The rank-based non-parametric analysis was used to check the rank-order of the three modalities (audio only, video only, visual music) and to determine if there were statistically significant differences between two (or more) modalities. For further investigation, a higher value in the mean rank indicates the upper rank; for example, in the “happy” clip group, A1 (Mean rank = 16.06) is higher in rank than the other two modalities, V1 (Mean rank = 10.06) and A1V1 (Mean rank = 11.38) on the evaluation index*.

* *Indicates a significant difference p < 0.05, while bold type indicates the highest rank per category*.

**Table 6:** **Visual music stimuli combination conditions information in the current study**.

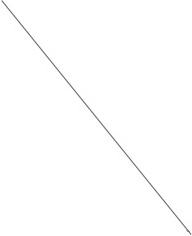		**Audio Only (Valence, Arousal)** **P = Positive, N = Negative, H = High, L = Low**				
		**AI** **(P, H)**	**A2** **(P, H)**	**A3** **(P, H)**	**A4** **(N, H)**	**A5** **(N, H)**
**Visual Only (Valence, Arousal)** **P = Positive, N = Negative, H = High, L = Low**	**V1** **(P, L)**	α 2(*C, I*)				β 3(*I, I*)
	**V2** **(P, L)**		α 2(*C, I*)		β 3(*I, I*)	
	**V3** **(P, H)**			α 1(C, C)^*^		
	**V4** **(N, H)**	β 2(*I, C*)			α 1(*C, C*)	
	**V5** **(N, H)**		β 2(*I, C*)			α 1*(C, C)*

Clip name and the emotional contexts (valence and arousal information) of each unimodal clip are indicated in bold types. Cross-binding codes indicate auditory-visual (A–V) combination conditions for all of the visual music stimuli in the current study. For example, β 2(I, C) where A2 combines with V5 indicates that it is an altered combination in contest condition with incongruent relationship in valence and congruent relationship in arousal between auditory and visual channels' emotional information. The emotional meanings were validated using similar evaluation and activity indices from the unimodal stimuli survey results in Experiment 1 and Experiment 2. α, original combination; β, altered combination; 1, Conformance; 2, Complement; 3, Contest;

* *, control*.

We performed a multivariate analysis of variance using a category as the independent variable “target-emotion” (three levels: happy, relaxed, and vigorous), with evaluation, activity, and potency indices as dependent variable. The test showed a statistically significant main effect of the clip (target-emotion) on the activity index [*F*_(2, 23)_ = 11.285, *p* = 0.000, $\eta_{p}^{2}$ = 0.518] and on the potency index [*F*_(2, 23)_ = 6.499, *p* = 0.006, $\eta_{p}^{2}$ = 0.382]. A *post-hoc* test indicated that clip 3, “vigorous,” showed significantly higher activity rating values than did clip 1 “happy” (*p* = 0.042) and clip 2 “relaxed” (*p* = 0.000). Clip 1, “happy,” showed significantly higher potency values than did clip 2, “relaxed” (*p* = 0.005). The means of the evaluation index shows that the clip “happy” had the highest evaluation value (*M* = 0.51, high-positive level), and the clip “relaxed” had the lowest (*M* = 0.12, mid-positive). The activity index in the visual music showed that the clip “vigorous” had the highest activity value (*M* = 0.51, high-active in arousal).

Further inspection of the perceived emotional information (evaluation, activity, and potency) on the interactions of “target emotion” and “modality” was conducted via a K–W test. The test results showed that there was no statistically significant difference among the three different modalities on the evaluation index. However, there were statistically significant differences on activity scores for the “happy” stimuli among the different modalities, $\chi_{\left(2\right)}^{2}$ = 11.409, *p* = 0.003, with a mean rank index score of 19.250 for A1, 10.130 for A1V1, and 8.130 for V1, and for the “vigorous” stimuli, χ2(2) = 6.143, *p* = 0.046, with a mean rank index score of 17.440 for A3, 9.190 for A3V3, and 10.880 for V3. Three statistically significant differences in potency scores were reported. The “happy” stimuli in different modalities showed $\chi_{\left(2\right)}^{2}$ = 6.244, *p* = 0.044, with a mean rank index score of 14.060 for A1, 15.880 for A1V1, and 7.560 for V1; the “relaxed” stimuli, $\chi_{\left(2\right)}^{2}$ = 6.687, *p* = 0.035, had a mean rank index score of 17.310 for A2, 8.250 for A2V2 and 11.940 for V2, while the “vigorous” stimuli showed $\chi_{\left(2\right)}^{2}$ = 8.551, *p* = 0.014, with a mean rank index of 6.560 for A3, 15.130 for A2V2, and 15.810 for V2 (Table 5).

##### EEG response

**Figure 4:**
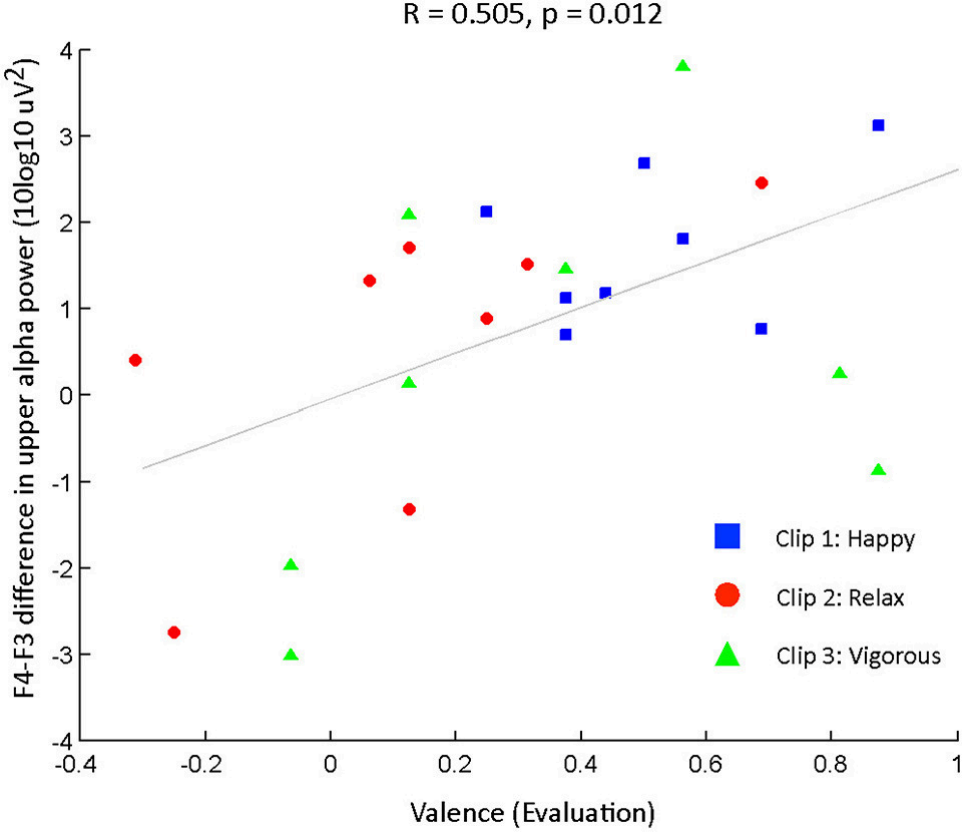
**Scatter plot and correlation between evaluation rating (valence) and upperAlpha frontal asymmetry**. Upper Alpha power was estimated for the early presentation of clips (10 s), and was log transformed (10log_10_ uV^2^). Frontal asymmetry was estimated as the difference of F3-F4 of log transform of upperAlpha band power [10log_10_(P)]. Pearson's correlation was used (*N* = 8 subjects).

**Figure 5:**
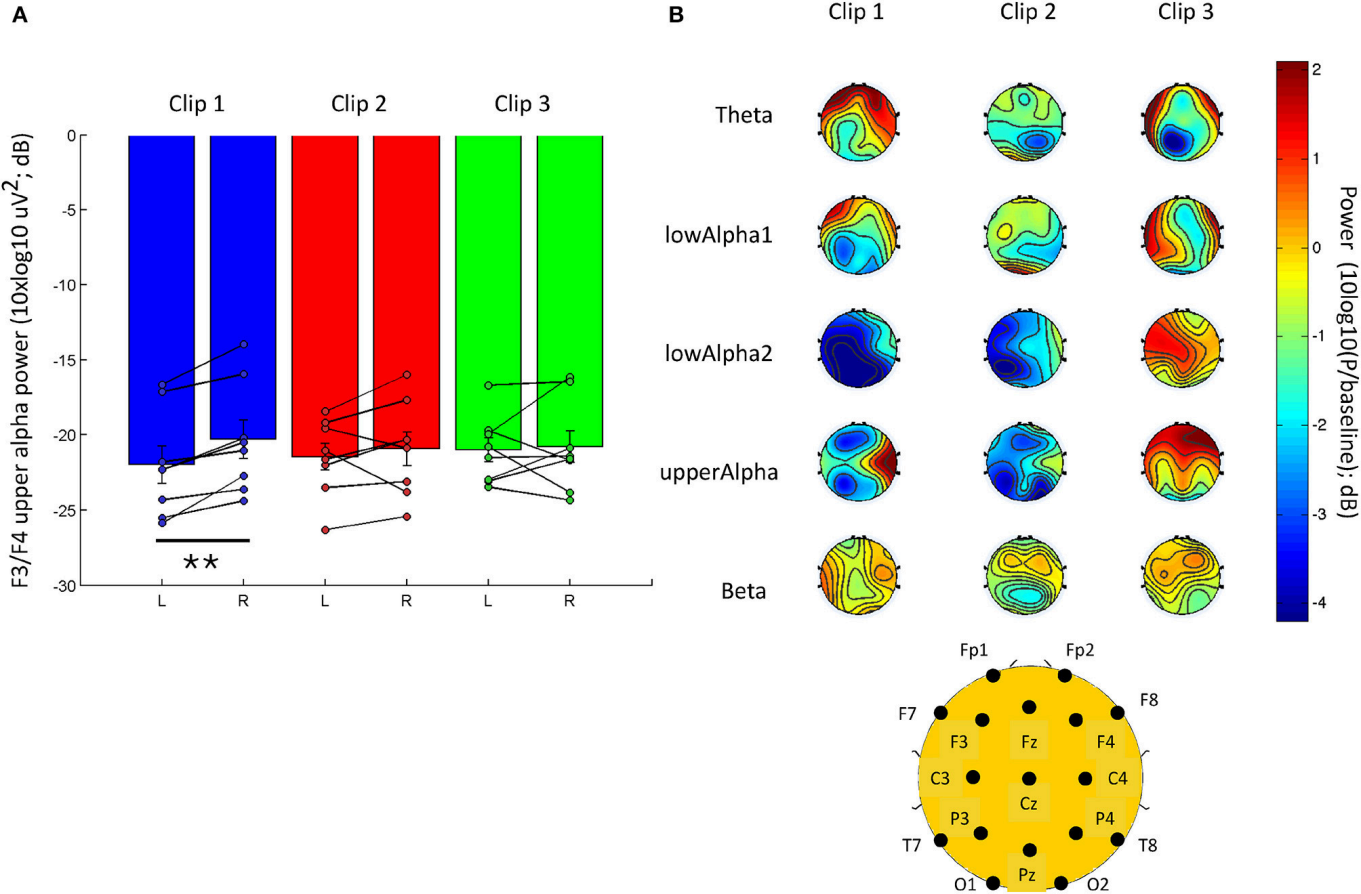
**EEG responses to each clip presentation. (A)** F3 and F4 frontal upper Alpha power (10log_10_ uV^2^) for the first 10 s of clip presentation for clip 1 (happy), clip 2 (relaxed), and clip 3 (vigorous). **(B)** The overall topographic EEG changes from the baseline for the early presentation of each clip (first 10 s). The power is normalized to the baseline (division) and log transformed (10^*^log10) to provide the baseline change in dB. See Figure S6 the full temporal dynamics of the EEG response to each clip. Data are presented on a scatter plot and mean ± s.e.m; ^**^ indicates a *p* ≤ 0.001.

The temporal dynamic of visual music creates complex responses in EEGs, with spatial, spectral, and temporal dependency for each clip (Figure S6). In order to remain within the scope of this study, we focused on a well-known EEG-based index of valence. It has been proposed that the frontal alpha asymmetry (EEG) could show close association with perceived valence in the early stage of the presentation of stimuli (Kline et al., 2000; Schmidt and Trainor, 2001; van Honk and Schutter, 2006; Winkler et al., 2010). We used the known neural correlates of emotion (frontal asymmetry in alpha power) to assess the internal responses of our subjects and to provide a partial physiological validation of our targeted-emotion elicitation from watching visual music. We found statistically significant correlations between evaluation and the frontal upperAlpha difference (evaluation-upperAlpha Subtraction: *r*_(24)_ = 0.505, *p* = 0.012; Figure 4). We also found a significant difference between F4 and F3 upperAlpha power [*t*_(7)_ = 5.2805; *p* = 0.001; paired *t*-test] for clip 1, the visual music stimulus showing the most positive response in valence. Clips 2 and 3 did not show significant differences in the frontal upperAlpha band power (Figure 5A).

In addition to the frontal asymmetry, we noted a qualitative average increase in frontal theta power and a decrease in power for lowAlpha2, compared to the baseline in the early presentation phase of clip 1 (first 10 s; Figure 5B). Subjects' responses during the early presentation of clip 2 (relaxed) exhibited a more symmetrical activity in the lower frequency bands (theta and lowAlpha1), as well as in beta power. Clip 3 (vigorous) elicited a stronger average increase in power. Notably, clip 3 showed more remarkable increase in frontal upperAlpha and lowAlpha2 power, compared to baseline recording.

### Discussion

In this experiment, we plotted the valence and arousal information of our designed clips on a well-known 2D plane with the means of the extracted evaluation and activity indices from psychometric self-rated subject surveys. The statistical analysis result indicates that our directive design of visual music delivered positive emotional meanings to the viewers with somewhat different valence, arousal, and texture information when compared to each other. The 2D plane illustration indicated that our constructed emotional visual music videos delivered positive emotional meanings to viewers that were similar to the intended target emotions. Comparisons of same target-emotional stimuli between modalities indicated that the likelihood of assessing valence (evaluation) information was similar for all three modality groups, while assessing the arousal (activity) and texture/control (potency) information was not similar. In other words, the target-emotion designs of audio, video, and visual music were likely to have similar information on valence levels with differences on arousal and texture/control levels. In addition, the frontal EEG asymmetry response during the early phase (first 10 s) of the visual music presentation correlated with the perceived valence index, which supports a possible relationship between valence ratings and the physiological response to positive emotion elicitation in our subjects. In our view, this preliminary experiment might provide a basis for the composition of synthetically integrated abstract visual music as unitary structural emotional stimuli that can elicit autonomic sensorial-perception brain processing; hence, we propose visual music as prototypic emotional stimuli for a cross-modal aesthetic perception study. The result of this experiment suggests the possibility of authenticating the continued investigation of aesthetic experiences using affective visual music stimuli as functions of information integration. However, the addition of negative emotional elements in experiment 2 seemed necessary to investigate how the auditory-visual integration condition might affect the overall aesthetic experience of visual music and the affective information interplay among modalities.

## Experiment 2: audio-visual integration experiment

To conduct the experiment, we first included two additional visual music stimuli created by a solo multimedia artist (who did not participate in the design of Experiment 1) within the negative spectrum of valence. We then confirmed the perceived emotional responses of participants watching our emotional visual music, and divided them into three indices, namely evaluation (valence), activity (arousal), and potency (texture/control). Lastly, we studied the audio-visual integration effect by cross-linking unimodal information from the original visual music to retain stimuli for all three integration conditions: conformance, complementation and contest (Section Stimuli Construction), and to investigate the enhancement effect of perceived emotion by synthetic cross-modal stimuli.

### Methods

To examine the association of different information-integration conditions between two modalities (audio and video) on aesthetic perception and the enhancement effect (to test our hypothesis regarding the integration enhancement effect), we conducted surveys involving audio-only, video-only, original visual music, and altered visual music stimuli. By using an analogous method in Experiment 1, we conducted psychometrical surveys of participants and assessed the evaluation, activity and potency factors across unimodal and cross-modal stimuli. We then compared the emotional information in unimodal channels and visual music, and checked for the presence of added values resulting from the A/V integration.

#### Participants

We recruited participants from the Dongah Institute of Media and Arts (DIMA) in Anseong, Korea, and Konyang University in Nonsan, South Korea for the four subject groups in our audio-visual integration experiment. Students from the DIMA enrolled in Home Recording Production or Video Production classes served as subjects in exchange for extra credit. Students from Konyang University enrolled in general education classes in Thought and Expression or Meditation Tourism served as voluntary subjects. Voluntary participants from Konyang University had the option of entering a draw to win a coffee voucher worth $10 in return for their participation. The groups were composed as follows:

- Audio-only group of 33 video production majoring students [mean age = 22.33, sex (m:f) = 10:23, minimum age = 21, maximum age = 25];
- Visual-only subjects of 27 students majoring in music [mean age = 25.15, sex (m:f) = 14:13, minimum = 21, maximum age = 28];
- The original visual music group was composed of 42 students majoring in cultural events and tourism [*mean age* = *22.50, sex (m:f)* = *22:20, minimum age* = *21, maximum age* = *29]*, and
- The altered visual music group was composed of 53 students who were studying majors that included fashion, optometry, tourism, business, computer engineering, economics, psychology, and the like [*mean age* = *22.32, sex (m:f)* = *24:29, minimum age* = *20, maximum age* = *27*].

Considering that our directive design stimuli were based on the concept of universal beauty in art, but that the survey subjects did not include participants form different cultures, the difference in the number of subjects in the groups was not critical (*n* ≥ 27 in all four groups). The survey kits and questionnaires were identical to the ones used in Experiment 1, except for the addition of a pleasant-unpleasant pair as a simple positive-negative affect response (see Figure S2). As in Experiment 1, subjects were fully informed, in both written and verbal forms, that they were participating in a survey for a study investigating aesthetic perception for scientific purposes. The consent of all subjects was obtained orally before taking part in the study, and the data were analyzed anonymously. Most of the visual music subjects (including both original and altered groups) were not taking majors related to music or visual art, with the exception of nine students (one digital content, one fashion design, two visual design, one cultural video production, and four interior design).

#### Stimuli construction

As briefly explained previously, we extracted the unimodal stimuli from five original visual music videos to divide them into independent audio-only and visual-only channel information (named A1–A5 and V1–V5, respectively). Based on the evaluated emotion from the unimodal information, we them cross-linked the clips to create altered visual music. This method was inspired by the three *basic models of multimedia* which is postulated by Cook (1998) and *combinations of visual and audio information based on the two-dimensional (2D) model* by Somsaman (2004). The result was nine cross-modal stimuli (five original and four altered clips) in three combinations of conditions: *conformance* (agreement between valence and arousal information from the video and audio channels), *complementation* (partial agreement between valence and arousal information from the video and audio channels) and *contest* (conflict between valence and arousal information from the video and audio channels) to use in our experiment (Figure S1).

#### Procedure

##### Unimodal

Surveys for the audio-only and visual-only groups were conducted on the same day at 11 a.m. and 1 p.m., and all the subjects in each group sat together in a large, rectangular audio studio classroom (~6 × 6.5 m) that was equipped with a wall-mounted screen (122 × 92 cm) A/V system and a pair of Mackie HRmk2 active monitor speakers. The audio-only group heard sound clips (60 s each) individually with a 20-s rest between presentations, ordered A2–A3–A4–A1–A5, and the visual-only group watched video clips (60 s each) that were ordered V2–V4–V3–V1–V5, with a 20-s rest between each presentation. Each student evaluated the emotional qualities of each stimulus by answering the questionnaires while listening to or watching the stimuli, and each session lasted ~10 min.

##### Cross-modal

We performed the psychometric rating experiments in the afternoon for each group at 1 and 3 p.m., and five visual music clips were played in each survey. All the subjects sat together in a general classroom (approximate room size 7 × 12 m) that was equipped with a wall-mounted, large-screen (305 × 229 cm) projection A/V system and a pair of Mackie SRM450 speakers. For the original visual music group, we played the stimuli in the order A4V4–A2V2–A5V5–A1V1–A3V3. For the altered visual music group, we played the stimuli in the order A5V1–A4V2–A1V4–A2V5–A3V3 (the control clip was the same as A3V3). Taking into account the temporal contextual changes that occur during the presentation of clips, the students responded the self-assessment questionnaires after watching each clip.

#### Data analysis

##### Reliability and competency tests of the indices

Before investigating the added value (enhancement effect) of cross-modal perception via visual music, we checked the reliability of our three emotional aspect factors (evaluation, activity, and potency) by using the data from all conditions in the experiment. The result showed a fairly high reliability value for the three indices (Cronbach's α = 0.728, *N* = 775), and the test indicated the omission of the activity index could lead to a higher Cronbach's alpha value (α = 0.915). The inter-relation correlation matrix values among the indices were evaluation-activity (0.295114), evaluation-potency (0.843460), and activity-potency (0.223417). A Pearson's correlation test indicated that three emotional indices were strongly associated: [evaluation-activity: *r*_(775)_ = 0.295, *p* < 0.000, evaluation-potency: *r*_(775)_ = 0.843, *p* < 0.000, and activity-potency: *r*_(775)_ = 0.223, *p* < 0.000]. An independent-sample *t*-test of three indices was conducted to compare the unimodal group and the cross-modal group in the evaluation, activity, and potency level conditions. The result indicated no significant differences between unimodal (*n* = 300) and cross-modal groups (*n* = 475), except in the activity values of unimodal (0.216 ± 0.313) and cross-modal (0.139 ± 0.279) conditions; *t*_(773)_ = 3.587, *p* = 0.000, *d* = 0.260. The results indicated higher mean activity values for audio only (0.342 ± 0.219) than for visual only (0.062 ± 0.341); *t*_(219, 71)_ = 8.267, *p* = 0.000, *d* = 0.975. In addition, we found higher mean activity values for the original group (0.189 ± 0.230) than for the altered group [0.099 ± 0.306); *t*_(473)_ = 3.528, *p* = 0.000, *d* = 0.333]. To further check the competency of the activity indices, we checked the responses to our control clip (A3V3) of the original (*n* = 42) and altered cross-modal (*n* = 53) survey groups. As Levene's test for equality of variances also revealed no significant differences in the two groups' assessments of the control clip, this provides some evidence that the equal variance assumption is satisfied on the univariate level. We found no statistically significant differences among groups of the three indices, as determined by a one-way ANOVA: evaluation [*F*_(1, 93)_ = 0.051, *p* = 0.822, *d* = 0.047], activity [*F*_(1, 93)_ = 0.061, *p* = 0.806, *d* = 0.045], and potency [*F*_(1, 93)_ = 0.942, *p* = 0.336, *d* = 0.199]. The descriptive statistical results are shown in Table 7 and Table S1.

**Table 7:** **Mean and Standard Deviation Comparison between Original and Altered Groups for Evaluation, Activity, and Potency Indices of the Control Clip (A3V3)**.

		***N***	**Mean**	**Std. Deviation**	**Std. Error**	**95% Confidence Interval for Mean**		**Minimum**	**Maximum**
						**Lower Bound**	**Upper Bound**		
Evaluation	Original group	42	0.226	0.306	0.047	0.131	0.322	−0.438	0.938
	Altered group	53	0.212	0.292	0.040	0.132	0.293	−0.500	0.875
	Total	95	0.218	0.297	0.030	0.158	0.279	−0.500	0.938
Activity	Original group	42	0.238	0.216	0.033	0.171	0.305	−0.313	0.750
	Altered group	53	0.251	0.284	0.039	0.173	0.330	−0.625	1.000
	Total	95	0.245	0.255	0.026	0.193	0.297	−0.625	1.000
Potency	Original group	42	0.109	0.373	0.058	−0.008	0.225	−0.938	0.938
	Altered group	53	0.179	0.335	0.046	0.087	0.272	−0.625	0.813
	Total	95	0.148	0.352	0.036	0.076	0.220	−0.938	0.938

##### Statistical analysis

To investigate the perception of emotion in the information-integration conditions (conformance, complementation, and contest) between two modalities (audio and video), we performed a separate one-way ANOVA analysis for each survey group (five audio only, five video only, five original video music, and five altered visual music). *Post-hoc* multiple comparisons of significant ANOVA results were then performed using the Bonferroni correction. Levene's test results for equality of variances were recorded when violated. All statistical tests were performed using the Statistical Package for Social Science (SPSS) version 23.0. The silhouette-clustering index was used as a measure of clustering. A score close to 1 indicates a compact and well-separated cluster, while a score close to −1 indicates a cluster with a large spread and/or poor separability. The silhouette analysis was performed using MATLAB® (Mathworks, Inc.).

### Results

#### Emotional meanings of clips

Tests of normality, sphericity (Mauchly's test of sphericity), equality of covariance matrices (Box's M), and multicollinearity (Pearson's correlation) for the three indices indicated that our data have violations in assumptions check for skewness, kurtosis, sphericity, equality of covariance, and correlations to validate the use of parametric ANOVA or MANOVA tests across three modalities (audio only, video only, and audio-visual). Hence, for comparisons across modalities, we opted for the non-parametric alternative, K–W test. In order to focus on the investigation of “information-integration conditions,” we report only critical results related to cross-modal stimuli in the manuscript, and other detailed investigations of multiple K–W tests and one-way MANOVA tests using three indices (evaluation, activity, potency) as dependent variables. “Modality' (audio only, video only, and cross modal), “clip” (A1, A2, V1, V2, A3V3, A5V1, and so on), “synchronization” (original vs. altered), or any interactions (of modality, clip, and synchronization) as independent variables will be provided separately (Table S2).

Table 8 shows the indices' values (evaluation, activity, and potency) for each mode of presentation, namely unimodal stimuli and the auditory-visual integrated stimuli. The valence values for each clip were found to have similar values to those of auditory stimuli in the original groups (A1V1, A2V2, and A3V3 had positive valence; in other words, the evaluation index > 0; A4V4 and A5V5 had negative valence, as the evaluation index < 0 with small difference in variance; A1V1 (0.66 ± 0.20), A2V2 (0.43 ± 0.27), A3V3 (0.23 ± 0.31), A4V4 (−0.42 ± 0.28), and A5V5 (−0.20 ± 0.33).

**Table 8:** **Means and STDs of three indices describing the emotional meanings of each clip stimulus in statistics: the highest ***absolute*** value in valence (evaluation) is indicated in bold type**.

**Modality**	**Indices**	**Clip 1**	**Clip 2**	**Clip 3**	**Clip 4**	**Clip 5**
Audio only	Evaluation	0.59 ± 0.25 (A1)	0.33 ± 0.21 (A2)	0.16 ± 0.23 (A3)	−0.30 ± 0.24 (A4)	−0.35 ± 0.26 (A5)
	Activity	0.29 ± 0.15 (A1)	0.18 ± 0.22 (A2)	0.50 ± 0.20 (A3)	0.45 ± 0.22 (A4)	0.29 ± 0.15 (A5)
	Potency	0.52 ± 0.24 (A1)	0.22 ± 0.27 (A2)	0.04 ± 0.29 (A3)	−0.49 ± 0.21 (A4)	−0.23 ± 0.28 (A5)
Visual only	Evaluation	0.40 ± 0.35 (V1)	0.10 ± 0.37 (V2)	**0.26** ± **0.31** (V3)	−0.21 ± 0.29 (V4)	−0.20 ± 0.40 (V5)
	Activity	−0.05 ± 0.30 (V1)	−0.17 ± 0.35 (V2)	0.21 ± 0.21 (V3)	0.24 ± 0.30 (V4)	0.09 ± 0.35 (V5)
	Potency	0.35 ± 0.34 (V1)	0.00 ± 0.33 (V2)	0.31 ± 0.37 (V3)	−0.28 ± 0.27 (V4)	−0.39 ± 0.37 (V5)
Original visual music	Evaluation	**0.66** ± **0.20** (A1V1)	**0.43** ± **0.27** (A2V2)	0.23 ± 0.31 (A3V3)	−**0.42** ± **0.28** (A4V4)	−0.20 ± 0.33 (A5V5)
	Activity	0.26 ± 0.15 (A1V1)	0.17 ± 0.25 (A2V2)	0.24 ± 0.22 (A3V3)	0.03 ± 0.27 (A4V4)	0.25 ± 0.16 (A5V5)
	Potency	0.51 ± 0.25 (A1V1)	0.37 ± 0.25 (A2V2)	0.11 ± 0.37 (A3V3)	−0.48 ± 0.31 (A4V4)	−0.27 ± 0.34 (A5V5)
Altered visual music	Evaluation	0.41 ± 0.37 (A1V4)	0.06 ± 0.32 (A2V5)	0.21 ± 0.29 (A3V3)	−0.36 ± 0.32 (A4V2)	−**0.41** ± **0.27** (A5V1)
	Activity	0.25 ± 0.20 (A1V4)	−0.01 ± 0.28 (A2V5)	0.25 ± 0.28 (A3V3)	−0.11 ± 0.23 (A4V2)	0.11 ± 0.35 (A5V1)
	Potency	0.28 ± 0.38 (A1V4)	0.07 ± 0.28 (A2V5)	0.18 ± 0.34 (A3V3)	−0.37 ± 0.25 (A4V2)	−0.37 ± 0.25 (A5V1)

*Data are described as mean ± SD*.

To inspect perceived emotional information (evaluation, activity, and potency) in interactions of “synchronization” (original vs. altered), “modality” (audio only, visual only, and cross modal), and “clip” (A1 ~ A5V1), we conducted several K–W tests. The test results showed that there were statistically significant differences among the conditions caused by different synchronization, modalities, and clips on evaluation, activity, and potency indices (all *p* ≤ 0.001, except *p* < 0.05 for clip 3 (for potency), clip 4 (for evaluation and potency), and clip 5 for evaluation and activity), except clip 3 for evaluation (*p* = 0.389) and clip 5 for potency (*p* = 0.069), as shown in Table 9.

Table 9**Results of the Kruskal-Wallis Test comparing the effect of clips with similar emotional meanings across modalities and synchronizations of the three perceived emotion assessment indices (evaluation, activity, and potency)**.

**Table:** 

	**Emotion Assessment**	***N***	**Mean**	**Std. Deviation**	**Min**	**Max**	**Percentiles**		
	**Indices**						**25th**	**50th (Median)**	**75th**
**DESCRIPTIVE STATISTICS (NPar TEST)**									
Clip 1 Group	Evaluation	155	0.515	0.321	−0.560	1.000	0.375	0.563	0.750
	Activity	155	0.209	0.233	−0.560	0.630	0.125	0.250	0.375
	Potency	155	0.404	0.328	−0.880	0.940	0.250	0.438	0.625
	Total	155	18.610	20.115	1.000	61.000	11.000	14.000	14.000
Clip 2 Group	Evaluation	155	0.224	0.335	−0.560	1.000	0.000	0.250	0.438
	Activity	155	0.050	0.303	−0.750	0.750	−0.125	0.063	0.250
	Potency	155	0.172	0.312	−0.630	0.940	−0.063	0.188	0.438
	Total	155	25.740	18.849	2.000	62.000	22.000	25.000	25.000
Clip 3 Group	Evaluation	155	0.214	0.286	−0.500	1.000	0.000	0.250	0.438
	Activity	155	0.293	0.259	−0.630	1.000	0.125	0.250	0.500
	Potency	155	0.153	0.351	−0.940	0.940	−0.063	0.188	0.375
	Total	155	32.180	18.717	3.000	63.000	33.000	34.000	34.000
Clip 4 Group	Evaluation	155	−0.340	0.294	−1.000	0.630	−0.563	−0.375	−0.125
	Activity	155	0.108	0.329	−0.750	0.880	−0.125	0.125	0.375
	Potency	154	−0.416	0.295	−0.940	0.810	−0.625	−0.438	−0.250
	Total	155	38.280	19.531	4.000	64.000	42.000	42.000	44.000
Clip 5 Group	Evaluation	154	−0.305	0.322	−1.000	0.560	−0.516	−0.313	−0.063
	Activity	155	0.185	0.282	−0.690	1.000	0.000	0.188	0.375
	Potency	154	−0.316	0.307	−1.000	0.560	−0.500	−0.344	−0.125
	Total	155	44.730	21.274	5.000	65.000	51.000	51.000	55.000

**Table:** 

	**Emotion Assessment Indices**	**Clip ID**	***N***	**Mean Rank**	**Chi-Square**	***df***	**Asymp. Sig**.	**Effect Size (η^2^)**
**RANKS (KRUSKAL-WALLIS TEST)**								
Clip 1 Group	Evaluation^**^	A1	33	87.110	18.206	3	0.000	0.118
		A1V1	42	**98.200**				
		A1V4	53	64.390				
		V1	27	62.170				
		Total	155					
	Activity^**^	A1	33	90.180	27.684	3	0.000	0.180
		A1V1	42	85.060				
		A1V4	53	85.640				
		V1	27	37.130				
		Total	155					
	Potency^**^	A1	33	92.890	16.127	3	0.001	0.105
		A1V1	42	92.520				
		A1V4	53	61.710				
		V1	27	69.190				
		Total	155					
Clip 2 Group	Evaluation^**^	A2	33	92.300	36.510	3	0.000	0.237
		A2V2	42	**105.670**				
		A2V5	53	55.020				
		V2	27	62.590				
		Total	155					
	Activity^**^	A2	33	97.790	26.277	3	0.000	0.171
		A2V2	42	93.810				
		A2V5	53	68.490				
		V2	27	47.890				
		Total	155					
	Potency^**^	A2	33	83.800	31.111	3	0.000	0.202
		A2V2	42	107.130				
		A2V5	53	62.240				
		V2	27	56.540				
		Total	155					
Clip 3 Group	Evaluation	A3	33	66.970	3.019	3	0.389	0.020
		A3V3	42	80.830				
		A3V3c	53	78.600				
		V3	27	**85.890**				
		Total	155					
	Activity^**^	A3	33	116.110	31.326	3	0.000	0.203
		A3V3	42	67.930				
		A3V3c	53	70.920				
		V3	27	60.980				
		Total	155					
	Potency^*^	A3	33	61.650	10.298	3	0.016	0.067
		A3V3	42	73.940				
		A3V3c	53	81.310				
		V3	27	97.800				
		Total	155					
Clip 4 Group	Evaluation^*^	A4	33	85.830	10.020	3	0.018	0.065
		A4V2	53	72.420				
		A4V4	42	66.180				
		V4	27	**97.760**				
		Total	155					
	Activity^**^	A4	33	124.860	66.513	3	0.000	0.432
		A4V2	53	48.070				
		A4V4	42	67.400				
		V4	27	95.960				
		Total	155					
	Potency^**^	A4	32	68.090	12.611	3	0.006	0.082
		A4V2	53	82.140				
		A4V4	42	64.350				
		V4	27	100.000				
		Total	154					
Clip 5 Group	Evaluation^*^	A5	32	71.410	13.786	3	0.003	0.090
		A5V1	53	62.760				
		A5V5	42	**94.370**				
		V5	27	87.410				
		Total	154					
	Activity^*^	A5	33	97.380	14.845	3	0.002	0.096
		A5V1	53	67.800				
		A5V5	42	86.980				
		V5	27	60.370				
		Total	155					
	Potency	A5	33	90.940	7.101	3	0.069	0.046
		A5V1	53	70.310				
		A5V5	42	83.520				
		V5	26	65.370				
		Total	154					

*The rank-based non-parametric analysis was used to examine the rank-order of four clips in a group (audio only, video only, original visual music vs. altered visual music), and to determine whether there were statistically significant differences between two (or more) modalities. A higher value in mean rank indicates that the upper rank (for example, for the clip group 1, A1V1 (Mean rank = 98.200) is higher in rank than are the other three clips A1(Mean rank = 87.110), V1 (Mean rank = 10.06) or A1V4 (Mean rank = 64.390) on the evaluation index*.

* *Indicates a significant difference p < 0.05*,

** *indicates p < 0.001, and bold type indicates the highest rank in valence (evaluation)*.

To further investigate the significant differences shown in Table 9, we conducted several other K–W tests among different modalities in the same clip groups (for example, A1 vs. V1, A1 vs. A1V1, A1 vs. A1V4, A1V1 vs. A1V4, and so on) However, in order to focus on investigating the added-value effects that result in affective enhancement, we only report the results of the comparison between a high-responsive unimodal (audio only) vs. original visual music (Table 10), and unimodal (audio only) vs. altered visual music (Table 11). The comparison between auditory-channel only and the original synchronization cross-modal stimuli results indicated a statistically significant difference in potency scores between A2 and A2V2, $\chi_{\left(1\right)}^{2}$ = 6.173, *p* = 0.013, with a mean rank index score of 30.970 for A1 and 43.520 for A1V1. The A3 and A3V3 comparison showed a significant difference in the activity index, $\chi_{\left(1\right)}^{2}$ = 23.984, *p* = 0.000, with a mean rank index score of 51.850 for A3 and 27.120 for A3V3. The A4 and A4V4 comparison showed a significant difference in the activity index, $\chi_{\left(1\right)}^{2}$ = 33.707, *p* = 0.000, with a mean rank index score of 54.440 for A4 and 25.080 for A4V4, while the A5 vs. A5V5 comparison indicated a significant difference in the evaluation index, $\chi_{\left(1\right)}^{2}$ = 5.791, *p* = 0.016, with a mean rank index score of 30.630 for A5 and 42.740 for A5V5. No statistically different effect of clips (A1 or A1V1) on any of three indices (evaluation, activity, and potency) was shown. In four out of five comparison cases, the cross-modal stimuli showed a higher mean rank over audio-only in the same group in the evaluation index; A1V1 (mean rank = 40.420) vs. A1 (mean rank = 34.920), A2V2 (mean rank = 41.850) vs. A2 (mean rank = 33.110), A3V3 (mean rank = 41.200) vs. A3 (mean rank = 33.920), and A5V5 (mean rank = 42.740) vs. A5 (mean rank = 30.630).

Table 10**Results of a Kruskal-Wallis Test comparing the effect of the emotional meaning of clips between the auditory modality and the cross-modality (original synchronization) on the three perceived emotion assessment indices (evaluation, activity, and potency)**.

**Table:** 

	**Emotion Assessment Indices**	***N***	**Mean**	**Std. Deviation**	**Min**	**Max**	**Percentiles**		
							**25th**	**50th (Median)**	**75th**
**DESCRIPTIVE STATISTICS (NPar TEST)**									
Clip 1 Group	Evaluation	75	0.632	0.226	−0.060	1.000	0.500	0.688	0.813
	Activity	75	0.275	0.148	−0.060	0.630	0.188	0.250	0.375
	Potency	75	0.513	0.242	−0.250	0.880	0.375	0.500	0.688
	Total	75	6.600	4.997	1.000	11.000	1.000	11.000	11.000
Clip 2 Group	Evaluation	75	0.388	0.250	−0.130	1.000	0.188	0.375	0.563
	Activity	75	0.173	0.235	−0.500	0.750	0.063	0.188	0.313
	Potency	75	0.307	0.268	−0.250	0.940	0.125	0.313	0.500
	Total	75	13.200	9.995	2.000	22.000	2.000	22.000	22.000
Clip 3 Group	Evaluation	75	0.197	0.275	−0.440	0.940	0.000	0.188	0.375
	Activity	75	0.352	0.245	−0.310	0.810	0.188	0.375	0.500
	Potency	75	0.079	0.340	−0.940	0.940	−0.125	0.063	0.313
	Total	75	19.800	14.992	3.000	33.000	3.000	33.000	33.000
Clip 4 Group	Evaluation	75	−0.369	0.268	−1.000	0.190	−0.500	−0.438	−0.188
	Activity	75	0.212	0.324	−0.630	0.810	0.000	0.250	0.500
	Potency	74	−0.487	0.268	−0.940	0.440	−0.688	−0.500	−0.375
	Total	75	26.400	19.989	4.000	44.000	4.000	44.000	44.000
Clip 5 Group	Evaluation	74	−0.267	0.308	−1.000	0.500	−0.500	−0.219	−0.063
	Activity	75	0.271	0.156	−0.130	0.560	0.188	0.250	0.375
	Potency	75	−0.254	0.313	−1.000	0.500	−0.500	−0.250	−0.063
	Total	75	33.000	24.986	5.000	55.000	5.000	55.000	55.000

**Table:** 

	**Emotion Assessment Indices**	**Clip ID**	***N***	**Mean Rank**	**Chi-Square**	***df***	**Asymp. Sig**.	**Effect Size (η^2^)**
**RANKS (KRUSKAL-WALLIS TEST)**								
Clip 1 Group	Evaluation	A1	33	34.920	1.183	1	0.277	0.016
		A1V1	42	**40.420**				
		Total	75					
	Activity	A1	33	39.650	0.344	1	0.558	0.005
		A1V1	42	36.700				
		Total	75					
	Potency	A1	33	38.060	0.000	1	0.983	0.000
		A1V1	42	37.950				
		Total	75					
Clip 2 Group	Evaluation	A2	33	33.110	2.994	1	0.084	0.040
		A2V2	42	**41.850**				
		Total	75					
	Activity	A2	33	39.200	0.180	1	0.672	0.002
		A2V2	42	37.060				
		Total	75					
	Potency^*^	A2	33	30.970	6.173	1	0.013	0.083
		A2V2	42	43.520				
		Total	75					
Clip 3 Group	Evaluation	A3	33	33.920	2.074	1	0.150	0.028
		A3V3	42	**41.200**				
		Total	75					
	Activity^**^	A3	33	51.850	23.984	1	0.000	0.324
		A3V3	42	27.120				
		Total	75					
	Potency	A3	33	34.800	1.274	1	0.259	0.017
		A3V3	42	40.510				
		Total	75					
Clip 4 Group	Evaluation^*^	A4	33	**43.670**	4.027	1	0.045	0.054
		A4V4	42	33.550				
		Total	75					
	Activity^**^	A4	33	54.440	33.707	1	0.000	0.456
		A4V4	42	25.080				
		Total	75					
	Potency	A4	32	38.660	0.164	1	0.685	0.002
		A4V4	42	36.620				
		Total	74					
Clip 5 Group	Evaluation^*^	A5	32	30.630	5.791	1	0.016	0.079
		A5V5	42	**42.740**				
		Total	74					
	Activity	A5	33	42.110	2.129	1	0.145	0.029
		A5V5	42	34.770				
		Total	75					
	Potency	A5	33	39.500	0.281	1	0.596	0.004
		A5V5	42	36.820				
		Total	75					

*The rank-based non-parametric analysis was used to examine the rank-order of the three modalities (audio only, video only, vs. visual music), and to determine whether there were statistically significant differences between two (or more) modalities. A higher value in mean rank indicates the upper rank (for example, in the clip group 1, A1V1 (Mean rank = 40.420) is higher in rank than is A1 (Mean rank = 34.920) on the evaluation index*.

* *Indicates significant difference p < 0.05*,

** *indicates p < 0.001, and bold type indicates the highest rank in valence (evaluation)*.

Table 11**Results of the Kruskal-Wallis Test comparing the effect of the emotional meaning of clips between auditory modality and cross-modality (altered synchronization) on the three perceived emotion assessment indices (evaluation, activity, and potency)**.

**Table:** 

	**Emotion Assessment Indices**	***N***	**Mean**	**Std. Deviation**	**Min**	**Max**	**Percentiles**		
							**25th**	**50th (Median)**	**75th**
**DESCRIPTIVE STATISTICS (NPar TEST)**									
Clip 1 Group	Evaluation	86	0.480	0.336	−0.560	1.000	0.375	0.563	0.688
	Activity	86	0.264	0.182	−0.500	0.630	0.188	0.281	0.375
	Potency	86	0.371	0.352	−0.880	0.940	0.188	0.438	0.625
	Total	86	9.010	6.359	1.000	14.000	1.000	14.000	14.000
Clip 2 Group	Evaluation	86	0.160	0.310	−0.560	0.880	−0.016	0.188	0.375
	Activity	86	0.064	0.275	−0.750	0.500	−0.125	0.125	0.313
	Potency	86	0.127	0.286	−0.560	0.940	−0.063	0.125	0.313
	Total	86	16.170	11.250	2.000	25.000	2.000	25.000	25.000
Clip 4 Group	Evaluation	86	−0.339	0.289	−0.940	0.630	−0.563	−0.375	−0.125
	Activity	86	0.108	0.352	−0.750	0.810	−0.188	0.031	0.438
	Potency	85	−0.423	0.288	−0.940	0.810	−0.563	−0.438	−0.313
	Total	86	27.420	18.587	4.000	42.000	4.000	42.000	42.000
Clip 5 Group	Evaluation	85	−0.389	0.262	−0.880	0.250	−0.594	−0.438	−0.188
	Activity	86	0.182	0.300	−0.690	0.810	−0.063	0.250	0.438
	Potency	86	−0.315	0.266	−1.000	0.440	−0.500	−0.313	−0.188
	Total	86	33.350	22.501	5.000	51.000	5.000	51.000	51.000

**Table:** 

	**Emotion Assessment Indices**	**Clip ID**	***N***	**Mean Rank**	**Chi-Square**	***df***	**Asymp. Sig**.	**Effect Size (η^2^)**
**RANKS (KRUSKAL-WALLIS TEST)**								
Clip 1 Group	Evaluation^*^	A1	33	**51.300**	5.267	1	0.022	0.062
		A1V4	53	38.640				
		Total	86					
	Activity	A1	33	44.620	0.110	1	0.740	0.001
		A1V4	53	42.800				
		Total	86					
	Potency^**^	A1	33	54.210	9.897	1	0.002	0.116
		A1V4	53	36.830				
		Total	86					
Clip 2 Group	Evaluation^**^	A2	33	**57.450**	16.803	1	0.000	0.198
		A2V5	53	34.810				
		Total	86					
	Activity^**^	A2	33	54.240	9.982	1	0.002	0.117
		A2V5	53	36.810				
		Total	86					
	Potency^*^	A2	33	51.470	5.502	1	0.019	0.065
		A2V5	53	38.540				
		Total	86					
Clip 4 Group	Evaluation	A4	33	**48.200**	1.905	1	0.168	0.022
		A4V2	53	40.580				
		Total	86					
	Activity^**^	A4	33	67.650	50.346	1	0.000	0.592
		A4V2	53	28.460				
		Total	86					
	Potency	A4	32	38.330	1.858	1	0.173	0.022
		A4V2	53	45.820				
		Total	85					
Clip 5 Group	Evaluation	A5	32	**46.410**	0.984	1	0.321	0.012
		A5V1	53	40.940				
		Total	85					
	Activity^*^	A5	33	52.480	6.994	1	0.008	0.082
		A5V1	53	37.910				
		Total	86					
	Potency^*^	A5	33	51.360	5.357	1	0.021	0.063
		A5V1	53	38.600				
		Total	86					

*The rank-based non-parametric analysis was used to examine the rank-order of the three modalities (audio only, video only, vs. visual music), and to determine whether there were statistically significant differences between two (or more) modalities. A higher value in mean rank indicates the upper rank; (for example, in the clip group 1, A1 (Mean rank = 51.300) is higher in rank than is A1V4 (Mean rank = 38.640) on the evaluation index*.

* *Indicates significant difference p < 0.05*,

** *indicates p < 0.001, and bold type indicates the highest rank per category*.

The results of the comparison between auditory-channel only and altered (cross-binding) synchronization cross-modal stimuli revealed eight (out of 12) cases of statistically significant differences on the evaluation, activity, and potency indices (Table 11). In all clip groups, the cross-modal stimuli showed a lower mean rank than audio-only in the same group on the evaluation index. A more detailed investigation of the effects of the individual clips on three indices was conducted via a one-way ANOVA, and the result thereof will be provided separately (Table S3).

**Figure 6:**
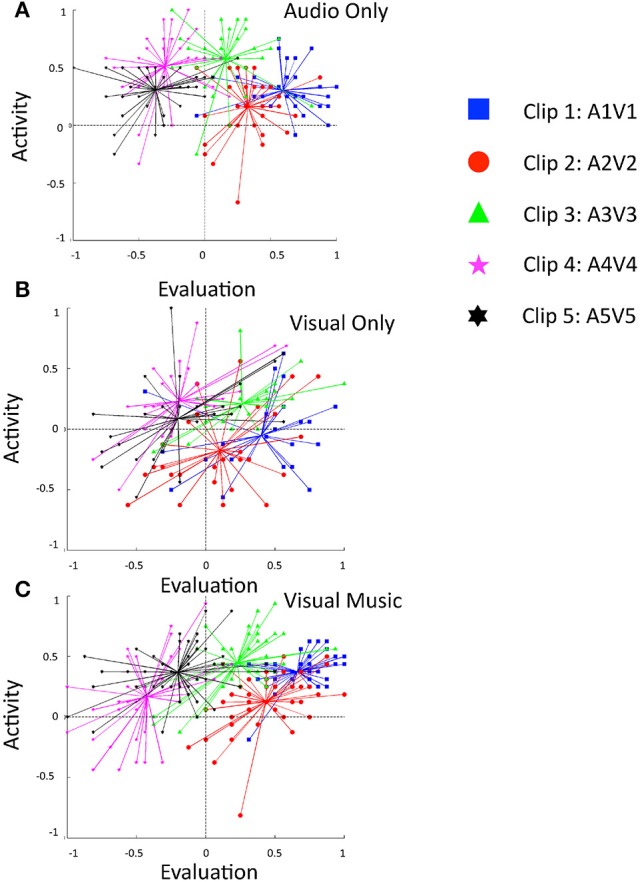
**Comparisons of immanent emotions in the three clip stimuli using the evaluation and activity indices (circumplex method) for the modalities. (A)** audio-only, **(B)** visual-only, and **(C)** visual music (cross-modal stimuli), represented by blue square (happy, clip 1), red disk (relaxed, clip 2) green triangle (vigorous, clip 3), pink star (clip 4, no target emotion), and black six-pointed star (clip 5, no target emotion). The symbols show the subjects' evaluations and activity index values for each clip.

**Figure 7:**
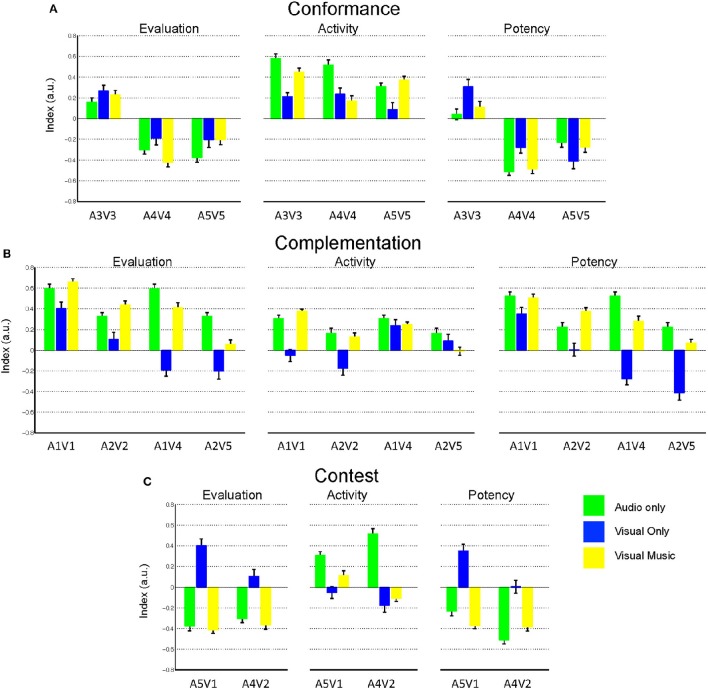
**Bar graph summary of the immanent emotion for the nine visual music stimuli using evaluation, activity, and potency for the combination conditions of (A) conformance**, **(B)** complementation and **(C)** contest. Data are shown as mean ± s.e.m. The mean increase of evaluation index was observed for clips A1V1 and A2V2 compared to their respective unimodal responses.

Similarly to Figure 3, the scatter plot representation of the original five visual music clips in 2D quadrants (valence and arousal dimensions) per modality (visual only, audio only, and visual music) is shown in Figure 6 as a simple representation of the overall emotion information derived from the clips. Emotional meaning aspects of each stimuli using the evaluation, activity and potency indices, and following the three media combination conditions (congruence, complementation, contest), are illustrated as bar graphs in Figure 7.

#### Compactness check

We used the silhouette index to quantify the quality of clustering for each mode of presentation within the 2D plane (Figure 6). For each clip, the silhouette value was obtained from each subject's response by using the evaluation and activity indices (Table 9), and returned a value representing the compactness (cohesion in emotional rating) and separation (differentiation from other clip responses) of the subjects' responses for different modality or different spatio-temporal combinations (such as separating original auditory-visual synchronizations into altered combinations) for each clip per modality.

The clustering analysis shown in Table 12 indicates that the audio only category (sil = 0.085) and original visual music category (sil = 0.045) were better clustered overall compared to the visual only category (sil = −0.076) and the altered visual music category (sil = −0.066). In particular, clip 1 showed the best clustering score of all the clips for the audio-only and visual music categories, indicating that its emotional connotation was best recognized by the subjects.

**Table 12:** **Silhouette clustering index values using 2D factors (evaluation and activity) rating indices for the data presented in Figure 6**.

	**Clip 1**	**Clip 2**	**Clip 3**	**Clip 4**	**Clip 5**	**Mean**
Audio Only	0.274048	−**0.044834**	0.177347	−0.101890	**0.123215**	**0.085577**
Visual Only	−0.103724	−0.172770	**0.209722**	−0.013282	−0.301105	−0.076232
Original Visual Music	**0.538540** (A1V1)	−0.134250 (A2V2)	−0.181865 (A3V3)	**0.053864** (A4V4)	−0.047418 (A5V5)	0.045774
Altered Visual Music	(A1V4) 0.047593	(A2V5) −0.192984	(A3V3)^*^−0.121569	(A4V2) 0.048313	(A5V1) −0.111249	−0.065979

*Silhouette values are given for individual clips and overall clustering quality. Silhouette values range within [−1, 1], with 1 indicating the best clustering and −1 the worst clustering. Bold values indicate the best clustering values relative to each clip (row-wise comparison)*.

* , control clip.

We then checked the compactness of each clip based on the average distances between the mean rating of each clip (centroid) and the subject rating using Euclidean distance and the indices evaluation, activity, and potency as dimensions; this estimation was performed for each clip and for each modality. The A1V1 and A2V2 visual music integrations were revealed to be the most compact scores (clustering and average distribution) in all other modal categories.

### Discussion

#### Enhancement effect hypothesis verification

We used three major indices (evaluation, activity, and potency) to thoroughly quantify the aesthetic/emotional meanings of abstract synthetic visual music clips in this study. To identify any proof of the enhancement effect, we looked for heightened mean or median scores for auditory-visual compared to other comparable unimodal scores for subjects' responses on the evaluation index because it is valence factor, which indicates the “likeness” characteristic of emotion. We also considered compactness via a clustering analysis and an average distance assessment to inspect the enhancement effect in relation to congruency in valence and arousal information by inspecting media combination conditions (congruence, complementation, and contest).

In our study, three clips were categorized in the congruence condition (A3V3, A4V4, and A5V5), four clips in complementation, and two clips in the contest condition (see Table 6, Figure 7, and Figure S1). These three clips exhibited no indication of higher emotional perceptive responses in both mean and median comparisons in the valence level (Tables 8, 10). However, with regard to the median rank, A3V3 and A5V5 showed higher mean ranks than did the other modalities (Table 10, bold type). With regard to clustering, only A4V4 had a higher score compared to other modalities (Table 12), and none of them showed good compactness (Table 13). None of these three clips ranked higher than did the other modalities for more than two of the four different assumptions (mean comparison, median rank comparison, clustering score, and compactness score) to check an enhancement effect. Therefore, our result seems to contradict our original hypothesis that the enhancement effect would be reliant on the congruent media combination condition in valence and arousal throughout our experiment results.

**Table 13:** **Compactness of response**.

	**Clip 1**	**Clip 2**	**Clip 3**	**Clip 4**	**Clip 5**	**All Clips**
Audio only	0.34628204	0.394233616	**0.40508022**	**0.391176521**	**0.390453802**	**0.38544524**
Visual only	0.499628207	0.555422101	0.437046321	0.464087266	0.576930643	0.506622908
Original visual music	**0.295774755** (A1V1)	**0.393163965** (A2V2)	0.468533303 (A3V3)^*^	0.479346853 (A4V4)	0.466745095 (A5V5)	0.420712794
Altered visual music	0.454134911 A1V4	0.44759437 (A2V5)	0.460930243 (A3V3)^*^	0.419427517 (A4V2)	0.448666851 (A5V1)	0.44759437

*Average distances to centroid using the evaluation, activity, and potency indices. The centroid for each clip was estimated first, and the average distance of each subject's response for the same clip was then estimated. Closer to zero indicates more compactness. Bold values indicate the closest compactness to relative centroid of each clip*.

* *, control clip*.

However, in this study, we found a great likelihood of valence and texture/control (evaluation and potency) information assessments of the visual music linked to the polarity of the auditory channel information in the contest conditions (Figure 7). Although the means and median ranks of evaluation, activity, and potency of visual music fluctuated between the corresponding indices' values of the two unimodal channels in most cases (Tables 8, 9, and Figure 7), we observed a few possible heightened emotional perception cases (for example, positive-augmentation or negative-diminishment on emotional aspect factors). “A1V1” showed a positively augmented mean value in the evaluation (valence), the highest median rank in the evaluation (valence), and the strongest compactness (both clustering and average distance) compared to its comparable unimodal clips (Tables 8, 9, 12, 13); “A2V2” showed a positively augmented mean value for evaluation, the highest media rank in the evaluation, and the smallest average distance (highest compactness) compared to unimodal stimuli. “A1V1” and “A2V2” both belong to the complementation combination condition with congruency in valence (positive) and incongruency in arousal [active (+) in audio and passive (−) in visual]. “A4V4,” in the conformance condition, showed more negative values in evaluation, and the highest clustering density (Tables 8, 9). “A5V1,” in a contest condition, showed more increased negative values in the evaluation and potency indices, but its clustering density and general mean-distance results indicated that perceived emotions were widespread compared to corresponding dual unimodal sources. For all of the other integrations, the visual music emotional perception evaluation rate showed various degrees in levels of integration formations (Figure 7). Overall, these results indicate that synchronizing audio and video information in the complementation combination condition could show instances of heightened perceptive emotion (enhancement effect) of multimodal perception in our study.

#### Cross-modal interplays

In our investigations, we observed a few notable results: First, we observed an *auditory dominant polarization* tendency, consistent with a few previous studies demonstrating auditory dominance over abstract vision in the temporal and perceptual processing of multimodalities (Marshall and Cohen, 1988; Repp and Penel, 2002; Somsaman, 2004). Since visual music is an abstract animation representing the purity of music by nature, it may be imperative for visual music to have audio channel information conveying stronger affective meanings (evaluation, activity, and potency values) via visual channel information. Nonetheless, the differences in emotional information on arousal (activity index) between the auditory and visual modalities left the auditory channel emotion information with a higher arousal value, hence dominating the visual channel and transferring affective meaning toward the overall emotional perception of cross-modal perception (the absolute mean value of activity for the auditory channel always showed a greater level than did the visual channel in *all* nine visual music presentations, and the mean rank of audio only was always higher than was visual only, as seen in Tables 8, 9).

Second, from the compactness response and silhouette analysis, we found that the overall perceptual grouping of “gestalt” (whole beauty) or “schema” (conceptual frameworks for expectations) in auditory-visual domains showed disperse responses as a result of the altered, cross-matched stimuli compared to the original integrations. This indicates the interplay of semantic and structural congruency (sharing temporal accent patterns) between auditory and visual information in forming the focus of attention in cross-modal perception as cognitive psychologists implied good gestalt principles (see Cohen, 2001, p. 260). In our finding, the spatiotemporal information in the arbitrary cross-matching could not assemble into good synchronous groupings in structural and semantic features with temporal cues and movements (e.g., tempo, directions, dynamic); hence, it impeded creating better interplay focus of attention compared to the original stimuli. In particular, the arbitrary integration by cross-matching audio and video channel information from different sources created semantic and structural asynchronous distraction in multisensory perceptional grouping thus resulting in “worse” aesthetic emotion (e.g., A5V1).

**Figure 8:**
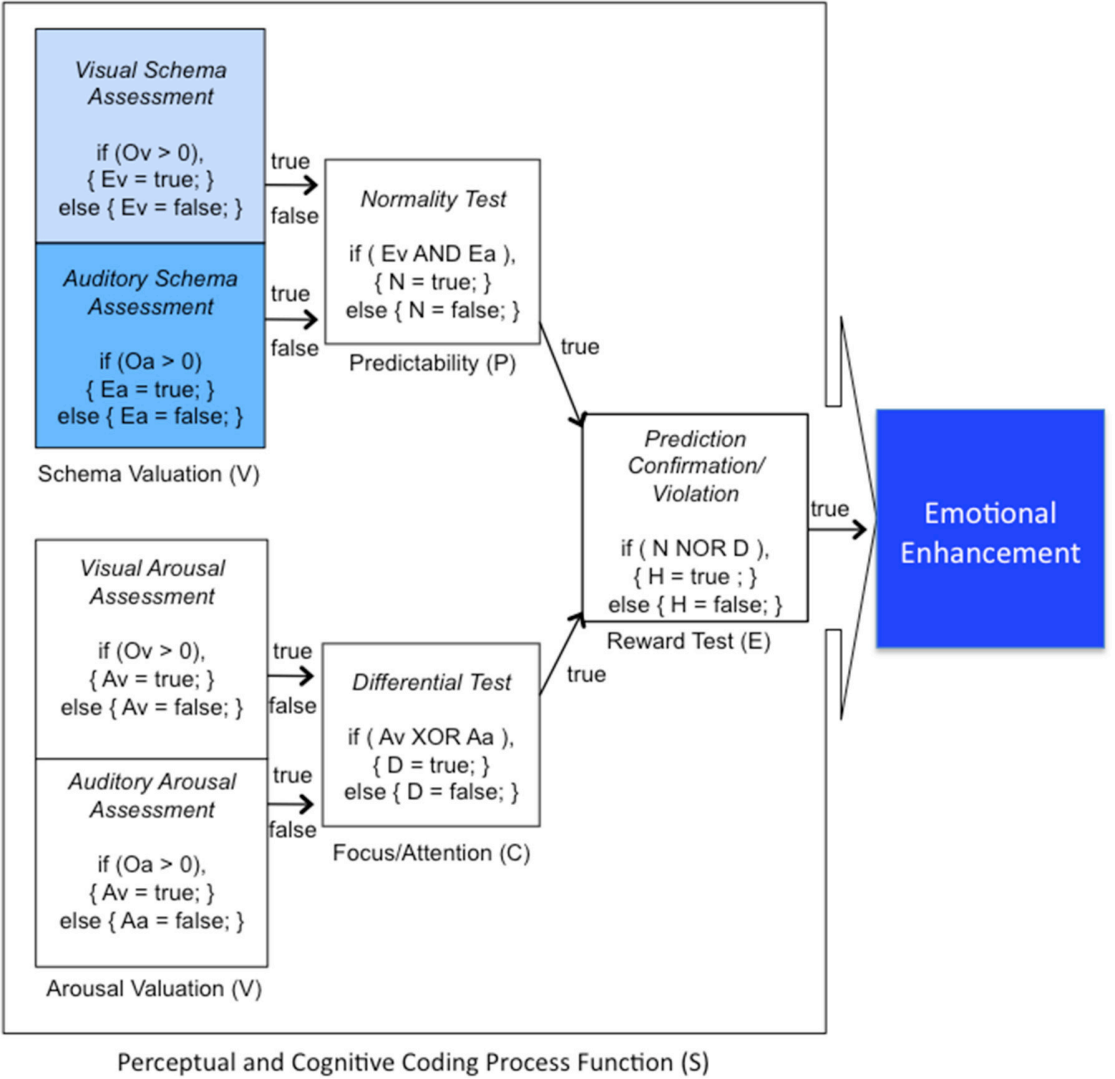
**Hypothesized conditional enhancement effects as added values in visual music**. The congruency in valence and texture/control aspects might stabilize the conjoint gestalt or schema to form good predictability (P) whereas the substantial differences in the arousal aspect between the dual modalities might emphasize focus/attention (C) that alter the continuous coding of predictive errors and recovering to predictable patterns for rewarding (E).

Finally, we found two cases of the positive enhancement effect in aesthetic perception resulting from the functions of information integration (auditory and visual) in this experiment, namely A1V1 and A2V2. They had contrary polarity for activity values [audio only activity (+) vs. visual only activity (−)], but evaluation (and potency) exhibited congruency in their polarity [all positive (+)]. This suggests that positive congruency in valence (and texture/control) information with uncompetitive discrepancy in arousal levels between the visual and audio channels might trigger the enhancement effect in aesthetic emotion appraisals (see Figure 8). This finding possibly relates to the art rewarding hypothesis which a state of uncertainty recovers into predictable patterns resulting to rewarding effect of increased expectedness (Van de Cruys and Wagemans, 2011). The congruency in valence and texture/control aspects in our A1V1 and A2V2 might, for example, implicitly stabilize the conjoint gestalt or schema that people use to form expectations or predictions whereas the substantial differences in the arousal aspect between the two modalities might implicitly emphasize elements that alter the continuous coding of predictive errors and recovering to predictable patterns. In other words, we assume that violations of prediction (predictive error) resulted from differences in any part where the two channels information return to a state of rewarding due to the formation of stable gestalt/schema. Other three original visual music stimuli did not indicate a strong enhancement effect in mean valence levels or median ranks although some showed improved compactness in silhouette index compared to its comparable unimodal stimuli (e.g., A4V4).

## General discussion

**Figure 9:**
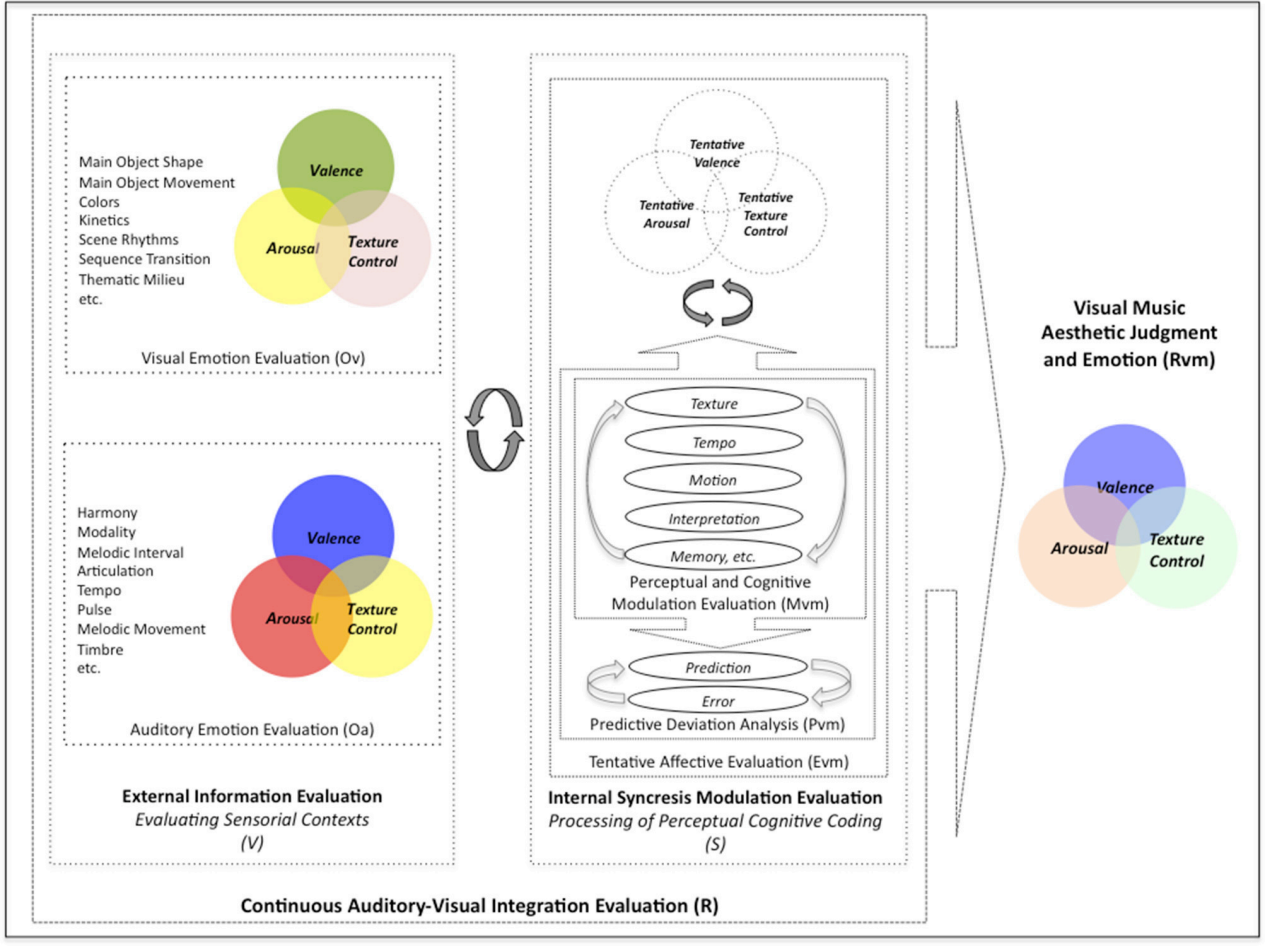
**A hypothesized functional-information-integration process within our model of continuous auditory-visual modulation integration perception**. Visual Music aesthetic experience requires multi-sensory perceptual, cognitive judgment, and processing that involve a number of continuous processing stages such as affective analysis (in valence, activity, and potency), perceptual and cognitive modulation analysis, and predictive deviation analysis.

Our study inspected the two unimodal channel interplays as a functional-information-integration apparatus by examining an enhancement effect in cross-modal evaluation processes of emotional visual music experience. During the two experiments, we could see that visual music can embody various structural variables that may cause important interactions between perceptual and cognitive aspects of the dual channels. The directive design guidelines we used to create target emotional stimuli in Experiment 1 (Tables 1, 2) indicate numerous parameters that can be used when creating visual music. The use of three extracted indices (evaluation, activity, and potency) to appraise ambiguous emotional meanings was effective to assess both the artwork and the subjects' responses in the study. It was also encouraging that we found a positive correlation between the evaluation index and the lateral frontal upperAlpha power differences in this study. Despite the small number of subjects in the study, the promising results from the frontal alpha asymmetry and its correlation with valence might encourage the inclusion of a wider range of physiological measurements to study the complex interactions between the external sensorial/perceptional context and the internal cognitive modulation/coding mechanism of aesthetic experience (see Figure 9 for an illustration of the partial potential interactions of sensorial context and cognitive coding, and Figure S5 for the temporal dynamics of spectral and spatial activation in EEG).

Our findings demonstrated the role of and interplays between the valence and arousal information in emotional evaluation of the auditory-visual aesthetic experience. The common effects of congruent positive valence between auditory and visual domains refer to good quality in gestalt/schema formation. Stronger arousal level of auditory channel information not only outweighed the visual channel information in making affective (valence and texture) judgments of visual music, but also ensued uncompetitive focus/attention to result increased states of positively predictable patterns. Hence, arousal levels and conditions hold a key role in modulating the excitement of affective emotion perception in visual music experience. Consequently, taking both dimensions of emotion (valence and arousal) into account is necessary to determine whether abstract auditory-visual stimuli carry strong, distinctive emotional meanings in particular.

As suggested by scholars in the field of aesthetic judgment and perception, emotion studies using works of art require particular insightful appraisal tools that should differ from depictive or prepositional expressions, such as pictures or languages (Takahashi, 1995). Hence, to infer emotional meanings from and to inspect the aesthetic perceptions of visual music as affective visual music, a careful choice of assessment factors for aesthetic perceptions of abstract visual music stimuli is crucial. More efforts to design refined and uncomplicated assessment apparatus for visual music perception would be challenging, yet worthy. The limitation of our mono-cultural background (college students in South Korea) makes it unable to generalize our findings as a truly representative universal aesthetic perception rule. Therefore, considering the findings in conjunction with all possible media integration conditions (Figure S1) will help to identify universal principles in visual music aesthetic perception. In addition, further investigations of how the interaction of three aspects of emotional meaning (valence, arousal, and texture/control) affect aesthetic emotion whilst considering temporal factors of visual music may also expand the understanding of the perceptual process in aesthetic experiences.

## Conclusion

There has been a significant development in theories and experiments that explain the process of aesthetic perception and experience during the past decade (for a review, see Leder and Nadal, 2014; nonetheless, research studies on emotion elicitation have long relied on inflexible, static, or non-intentionally designed uni/cross-modal stimuli. Due to the lack of sufficient research evidence, aesthetic researchers have been calling for more sophisticated investigations of the interplay of perceptual and cognitive challenges with using novel, complex, and multidimensional stimuli (Leder et al., 2004; Redies, 2015). The need for new empirical approaches in aesthetic science requires an extensive amount of principled research effort to study the numerous components of emotion and competencies via several measurements, as Scherer and Zentner (2001) explained. The investigations of the process whereby art evokes emotion using a novel attempt in cross-modal aesthetic studies hence necessitate extensive research efforts with certain measurement competencies as important aspects.

Our empirical study of audio-visual aesthetic perception has a cross-disciplinary approach including music, visual aspects, aesthetics, neuroscience, and psychology and takes more of a holistic than an elementary approach; this is unconventional in several ways when compared to classical, disciplined paradigms. Initially, strong demands from commercial industries for practical psychotherapeutic contents cued our research team to bring artistic issues into the science laboratory. It inspired us to create artwork with verified, literature-based correlations with positive emotions, and to find ways to validate the ambiguous nature of visual music via the observable assessment of suitable measurements to translate it into psychological and cognitive science investigations. The aesthetic experience is known to have three components (artist, artwork, and beholder), and our study involved all three aspects; however, psychological aesthetic studies have historically been related to how art evokes an emotional response from viewers instead of exploring the factors that motivate individuals to produce art (see Shimamura, 2012, p. 24). When we demonstrated a basis for the directive production of emotional visual music to our artists, they understandably complied with the intention of the emotional stimuli production (creating target-emotion eliciting visual music), and took the directive settings of the structural/formal components of visual stimuli and music (see Tables 2, 3) into account in their artistic activity (which involves well-developed and highly complex cognitive processes). Hence, we can claim that our emotional visual music stimuli constitute at least three predominant approaches in experimental aesthetic theories—expressionist, contextual, and formalist. Several theories have suggested models that explain human aesthetic perception and judgment processes (Leder et al., 2004; Chatterjee and Vartanian, 2014; Leder and Nadal, 2014; Redies, 2015), calling for more diverse empirical investigations that adopt various kinds of approaches. However, using real artwork in empirical research has generated disappointing results, although it is an interesting topic for artists and psychologists, and there is a need to extend previous approaches in emotional aesthetics to understand hedonic properties, cognitive operations, and greater compositional potential (for a review, see Leder et al., 2004). However, through our study, we believe that we have determined that the composition of abstract visual clips with directive design could cover a range of emotions, which can be assessed by evaluation, activity, and potency indices, and has the potential to be used as stimuli for more complex continuous response measures. Hence, we posit that properly controlled, well-designed visual music stimuli may be useful for future psychological and cognitive research studying the continuous reciprocal links between affective experience and cognitive processing, and specifically to understand how collective abstract expressions stimulate a holistic experience for audiences. In particular, because visual music has temporal narratives, it could be useful for future research to inspect the temporal dynamics of brain activity, skin conductance responses, changes in respiration or skin temperature as objective (autonomic) measures of emotional experiences in holistic information processing of the subject's state in relation to auditory and visual perception property controls. If possible, constructing a database of visual music with emotional meanings that provides a standardized set of abstractive auditory visual stimuli with accessible controls of various contextual parameters might be beneficial for future aesthetic emotion and aesthetic appreciation studies. The use of validated holistic stimuli and structural property controls may allow for investigations of integration synthesizing functions with semantic and syntax processing in auditory-visual aesthetic evaluation mechanisms.

To the best of our knowledge, our study is the first to propose a paradigm for the composition of abstract visual music with emotional validation at the unimodal, cross-modal, psychological and neurophysiological levels. Based on the findings of this study, we suggest that controlled, affective visual music can be a useful tool for investigating cognitive processing in affective aesthetic appraisals.

## Ethics statement

The study was approved by the Institutional Review Board of Korea Advanced Institute of Science and Technology. All our subjects were fully informed that they were participating in a survey for a study investigating aesthetic perception for a scientific research in both written and verbal forms. All participants signed a written informed consent form prior to engaging in the experiment.

## Author contributions

The conception or design of the work: IL, CL, and JJ. The acquisition, analysis, or interpretation of data for the work: IL and CL. Drafting the work: IL. Revising it critically for important intellectual content: IL and CL. Final approval of the version to be published: IL, CL, and JJ. Supervising the work overall: JJ. Agreement to be accountable for all aspects of the work in ensuring that questions related to the accuracy or integrity of any part of the work are appropriately investigated and resolved: IL, CL, and JJ.

## Funding

The research and creation of the abstract visual music (positive) contents used in this study were financed by the company Amore Pacific Corporation, 181 Hanggangro-2-ga, Yongsan-gu, Seoul, South Korea. The financial support included the research staff's remuneration, artists' remuneration, other technical facilities for the creation of multimedia contents and electrophysiological recordings subjects remuneration.

### Conflict of interest statement

The authors declare that the research was conducted in the absence of any commercial or financial relationships that could be construed as a potential conflict of interest.

## Supplementary material

Figure S1 **Possible situations in three (conformance, complementation, and contest) conditions of cross-modal combination**.

Figure S2 **Target emotion characteristics on the 2D (valence and arousal) plane illustration**.

Figure S3 **Survey questionnaire with 13 pairs of bipolar sensorial adjectives**. The questionnaire was initially designed in US English and was presented to the participants in the Korean language. The unpleasant-pleasant pair was only additional in experiment 2 surveys.

Figure S4 **Experiment design for stimuli clip presentation and rating**.

Figure S5 **Composition of the three emotional aspect indices (evaluation, activity, and potency) and the method of rating conversion**. A total of 12 pairs of bipolar ratings were used to extract the evaluation, activity, and potency indices. The indices were rescaled from the nine-point scales to a range of [−1, 1] as shown.

Figure S6 **Temporal and topographic responses to visual music presentations**. Average normalized power for 10-s non-overlapping epochs during the presentation of clip 1(a), clip 2(b), and clip 3(c)'s visual music. EEG power is estimated using a FFT method for each 10-s non-overlapping periods from baseline to end of clip presentation. The two first epochs (20 s) are averaged to provide a baseline power for each frequency range (theta, lowApha1, lowAlpha2, UpperAlpha, and Beta); all power values are then normalized according to baseline power and 10log10 transform (dB). The topographic positioning of EEG leads is shown in the inset (bottom right corner). Time (bottom label) indicates the center of the 10-s epoch. The dashed line represents the start of the clip presentation after baseline resting.

Table S1 **Independent ***T***-test result of two groups' responses to the control clip**.

Table S2 **Unreported results of K-W test across modalities results**.

Table S3 **One-way ANOVA analysis results for comparing clips within the same modality**.
