# Supplementary figures and images for: Arousal Rules: An Empirical Investigation into the Aesthetic Experience of Cross-Modal Perception with Emotional Visual Music
Source: Front Psychol. 2017 Apr 4;8:440. doi: 10.3389/fpsyg.2017.00440 (PMC5379063; doi:10.3389/fpsyg.2017.00440)

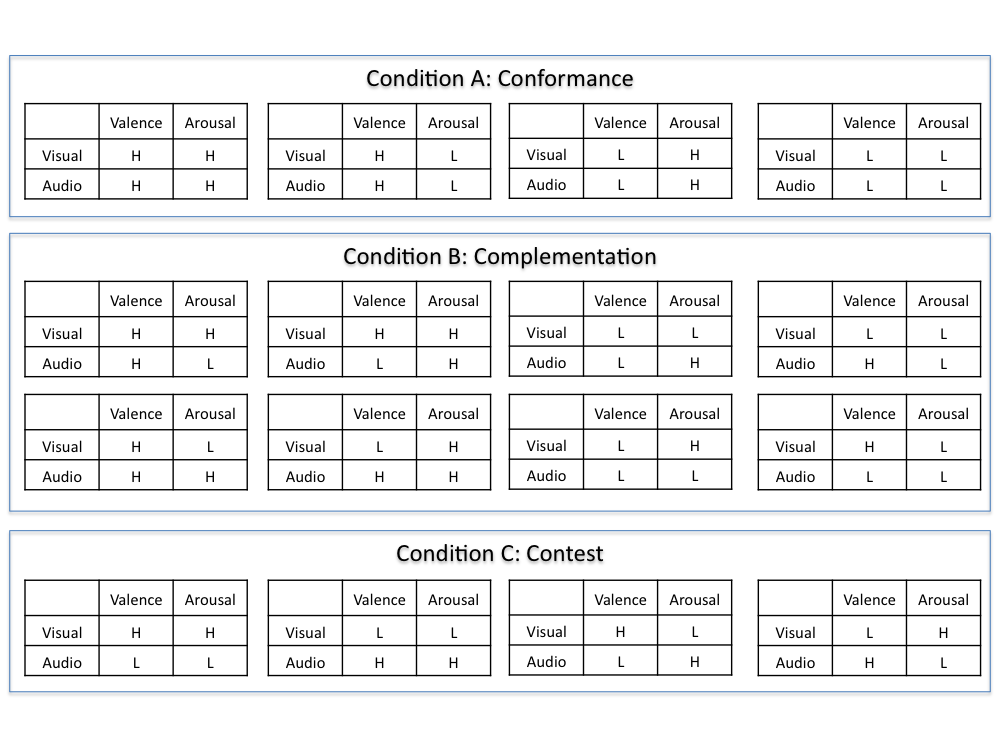

Supplement: Figure S1 — Possible situations in three (conformance, complementation, and contest) conditions of cross-modal combination. [file Image1.tif]

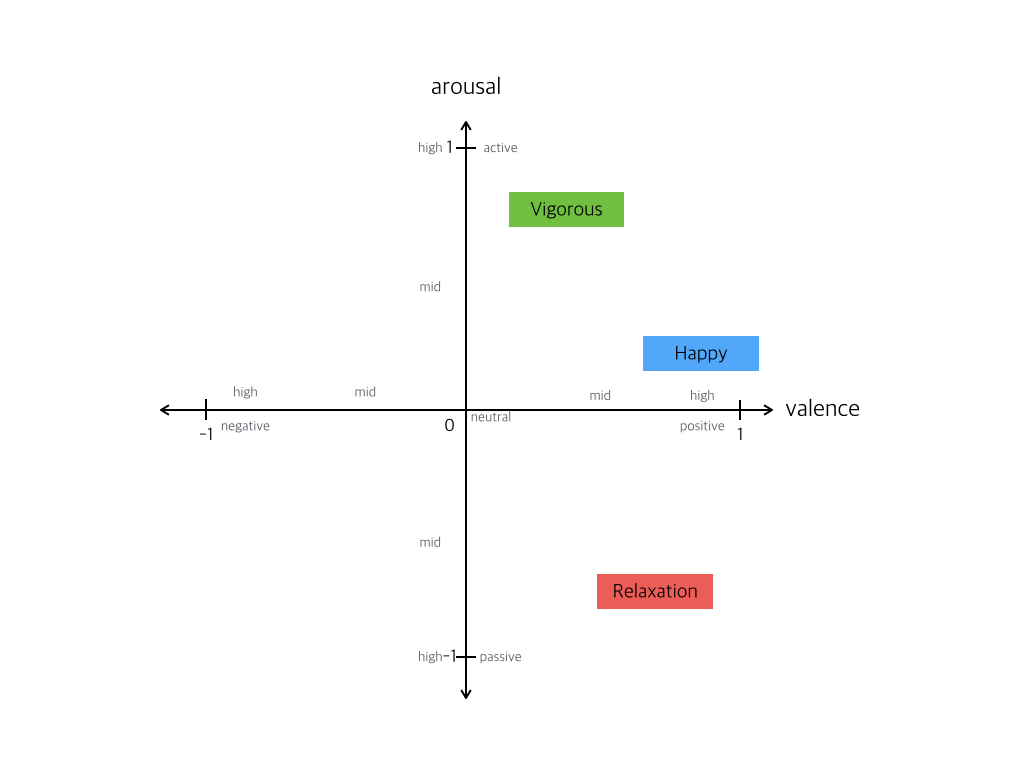

Supplement: Figure S2 — Target emotion characteristics on the 2D (valence and arousal) plane illustration. [file Image2.tiff]

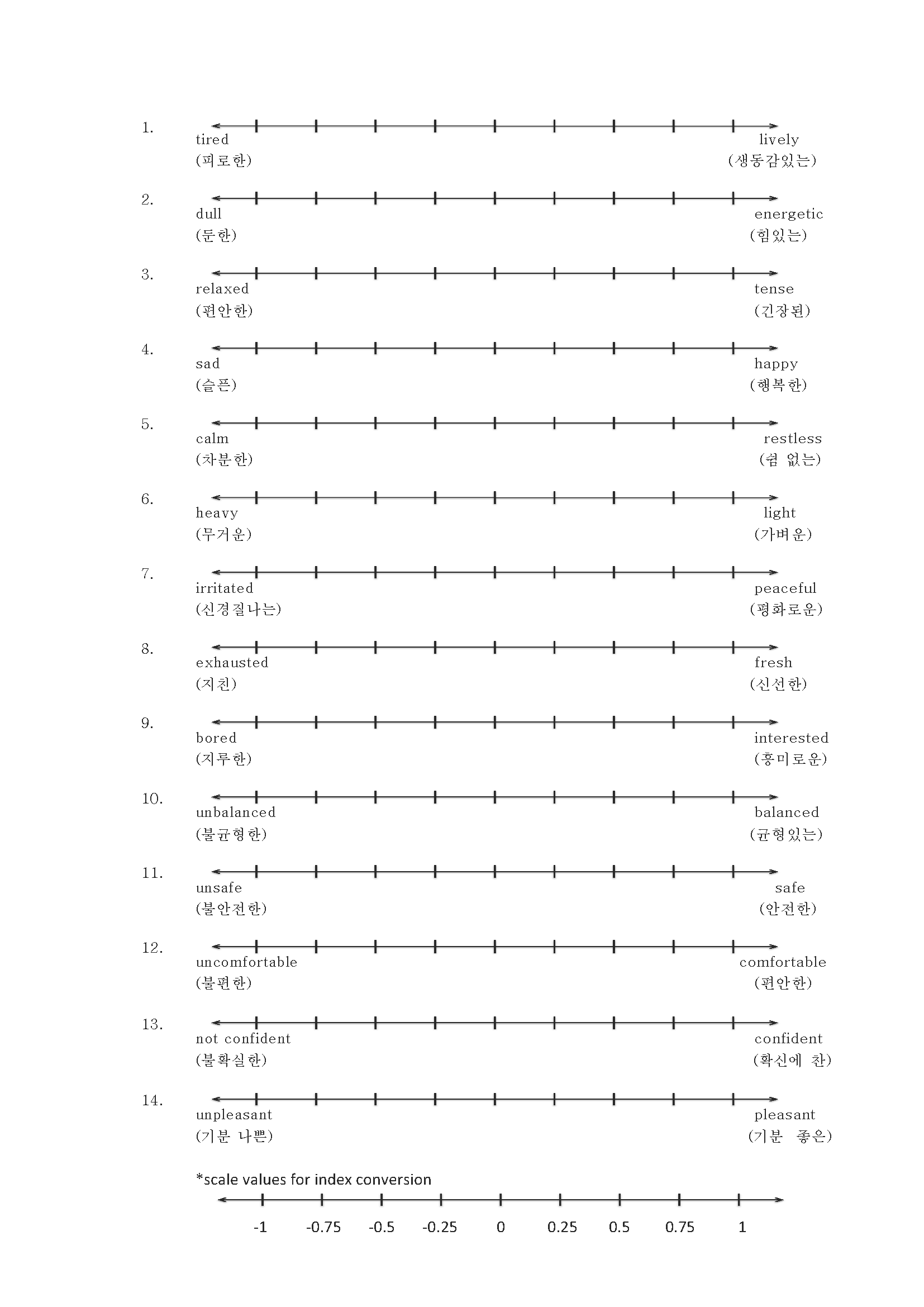

Supplement: Figure S3 — Survey questionnaire with 13 pairs of bipolar sensorial adjectives. The questionnaire was initially designed in US English and was presented to the participants in the Korean language. The unpleasant-pleasant pair was only additional in experiment 2 surveys. [file Image3.tiff]

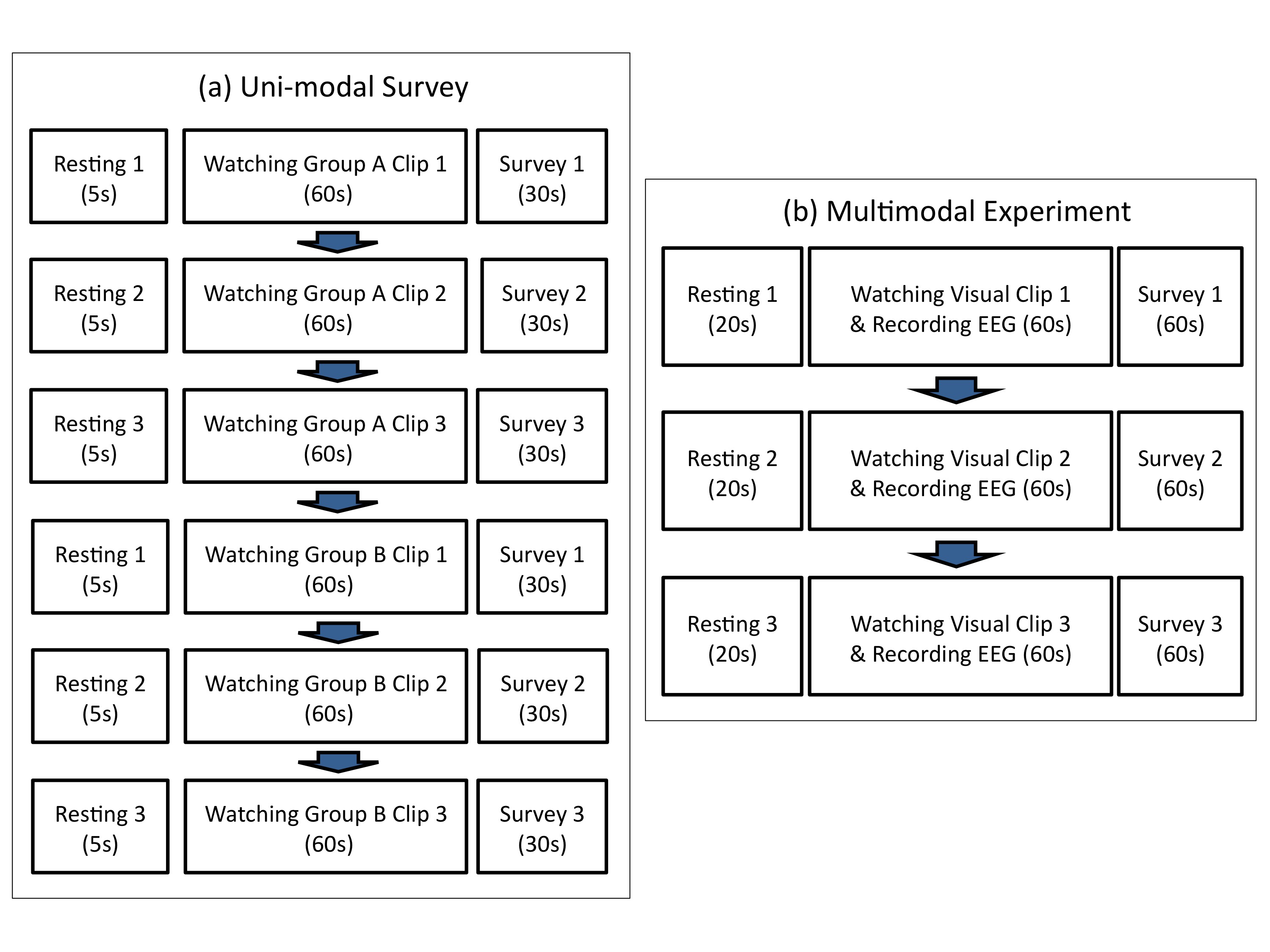

Supplement: Figure S4 — Experiment design for stimuli clip presentation and rating. [file Image4.jpg]

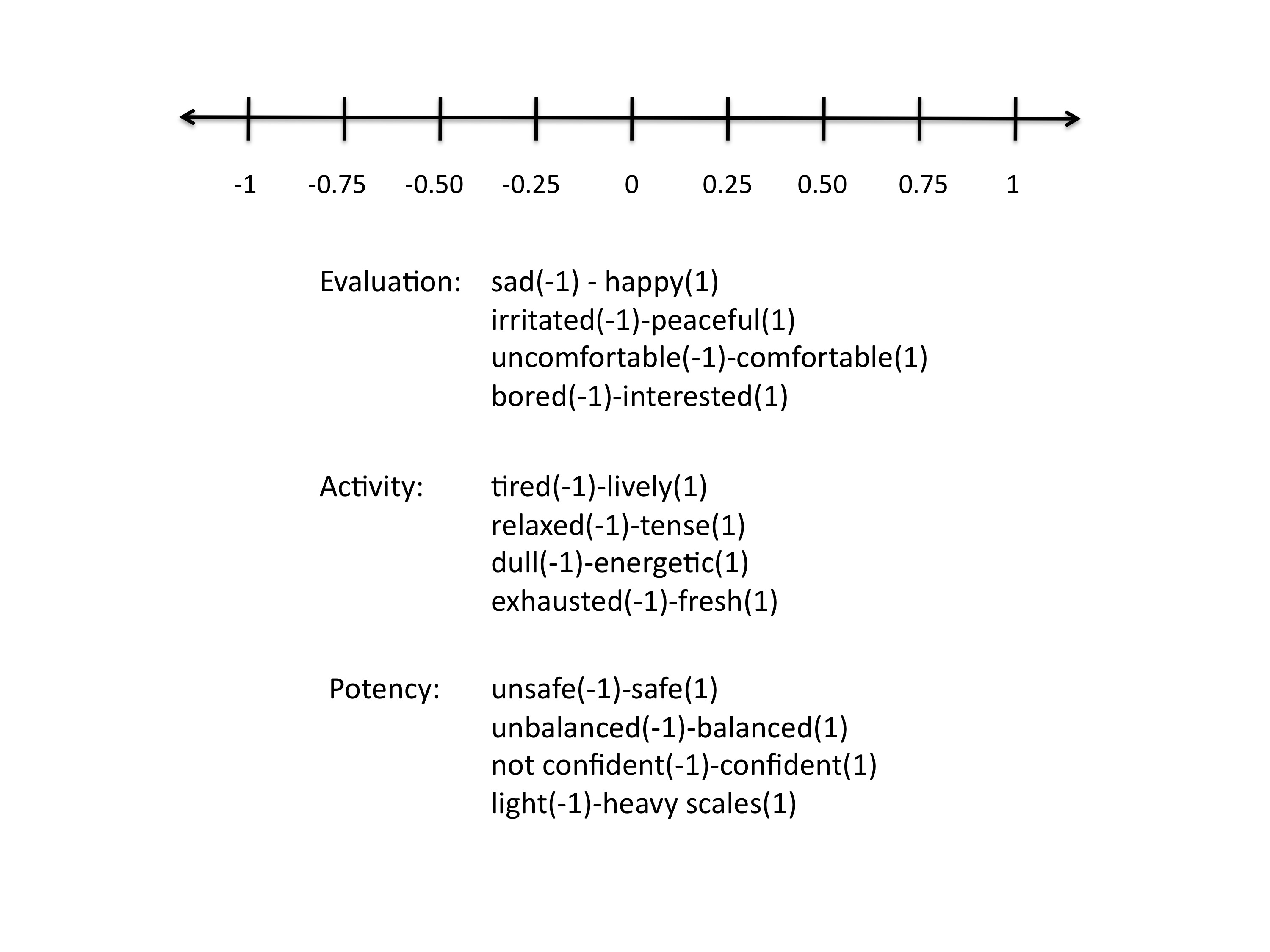

Supplement: Figure S5 — Composition of the three emotional aspect indices (evaluation, activity, and potency) and the method of rating conversion. A total of 12 pairs of bipolar ratings were used to extract the evaluation, activity, and potency indices. The indices were rescaled from the nine-point scales to a range of [−1, 1] as shown. [file Image5.jpg]

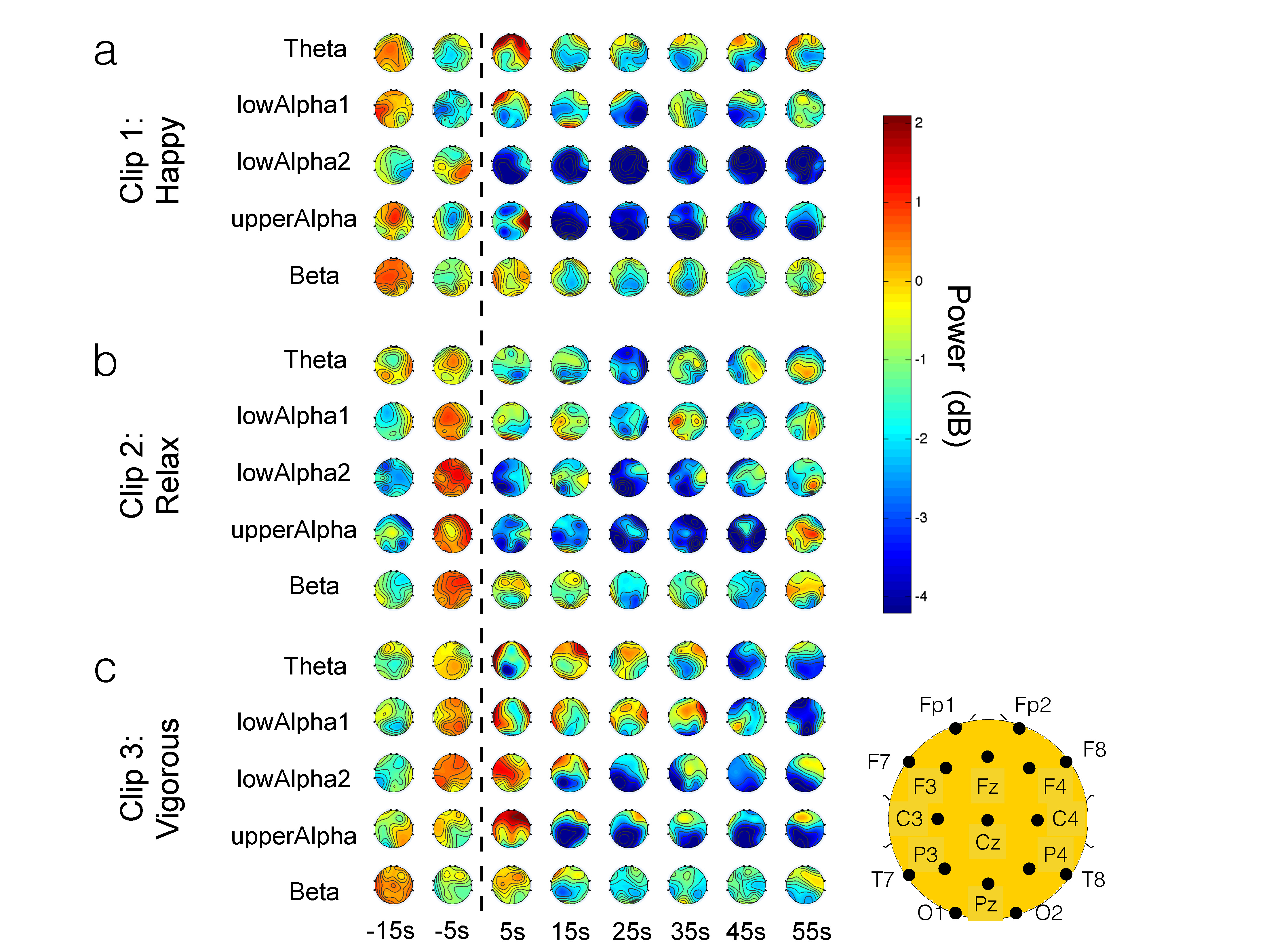

Supplement: Figure S6 — Temporal and topographic responses to visual music presentations. Average normalized power for 10-s non-overlapping epochs during the presentation of clip 1(a), clip 2(b), and clip 3(c)'s visual music. EEG power is estimated using a FFT method for each 10-s non-overlapping periods from baseline to end of clip presentation. The two first epochs (20 s) are averaged to provide a baseline power for each frequency range (theta, lowApha1, lowAlpha2, UpperAlpha, and Beta); all power values are then normalized according to baseline power and 10log10 transform (dB). The topographic positioning of EEG leads is shown in the inset (bottom right corner). Time (bottom label) indicates the center of the 10-s epoch. The dashed line represents the start of the clip presentation after baseline resting. [file Image6.jpg]
