# Supplementary material for: Arousal Rules: An Empirical Investigation into the Aesthetic Experience of Cross-Modal Perception with Emotional Visual Music
Source: Front Psychol. 2017 Apr 4;8:440. doi: 10.3389/fpsyg.2017.00440 (PMC5379063; doi:10.3389/fpsyg.2017.00440)
Supplement: Table S1 — Independent T-test result of two groups' responses to the control clip. [file Table1.PDF]

Oneway: Responses Comparison of Control Movie (A3V3)

Descriptives

		N	Mean	Std. Deviation	Std. Error	95% Confidence Interval for Mean		Minimum	Maximum
						Lower Bound	Upper Bound		
evaluaion	OriginalVisualMusic	42	.22619	.305860	.047195	.13088	.32150	-.438	.938
	AlteredVisualMusic	53	.21226	.291697	.040068	.13186	.29267	-.500	.875
	Total	95	.21842	.296516	.030422	.15802	.27882	-.500	.938
activity	OriginalVisualMusic	42	.23810	.216171	.033356	.17073	.30546	-.313	.750
	AlteredVisualMusic	53	.25118	.284435	.039070	.17278	.32958	-.625	1.000
	Total	95	.24539	.255303	.026194	.19339	.29740	-.625	1.000
potency	OriginalVisualMusic	42	.10863	.372977	.057552	-.00760	.22486	-.938	.938
	AlteredVisualMusic	53	.17925	.334791	.045987	.08697	.27153	-.625	.813
	Total	95	.14803	.352028	.036117	.07631	.21974	-.938	.938

Test of Homogeneity of Variances

	Levene Statistic	df1	df2	Sig.
evaluaion	.120	1	93	.730
activity	2.337	1	93	.130
potency	.320	1	93	.573

ANOVA

		Sum of Squares	df	Mean Square	F	Sig.
evaluaion	Between Groups	.005	1	.005	.051	.822
	Within Groups	8.260	93	.089		
	Total	8.265	94			
activity	Between Groups	.004	1	.004	.061	.806
	Within Groups	6.123	93	.066		
	Total	6.127	94			
potency	Between Groups	.117	1	.117	.942	.334
	Within Groups	11.532	93	.124		
	Total	11.649	94			
