# Supplementary material for: Arousal Rules: An Empirical Investigation into the Aesthetic Experience of Cross-Modal Perception with Emotional Visual Music
Source: Front Psychol. 2017 Apr 4;8:440. doi: 10.3389/fpsyg.2017.00440 (PMC5379063; doi:10.3389/fpsyg.2017.00440)
Supplement: Table S2 — Unreported results of K-W test across modalities results. [file Table2.PDF]

NPar Tests

Descriptive Statistics

	N	Mean	Std. Deviation	Minimum	Maximum	Percentiles		
						25th	50th (Median)	75th
Evaluation	60	.5073	.31220	-.44	1.00	.3750	.5625	.6875
Activity	60	.1375	.28463	-.56	.63	-.0469	.1875	.3125
Potency	60	.4437	.29912	-.38	.88	.2500	.4688	.6250
A1~A5V1	60	28.00	30.102	1	61	1.00	1.00	61.00

Kruskal-Wallis Test

Ranks

	A1~A5V1	N	Mean Rank
Evaluation	A1	33	34.88
	V1	27	25.15
	Total	60	
Activity	A1	33	39.91
	V1	27	19.00
	Total	60	
Potency	A1	33	34.62
	V1	27	25.46
	Total	60	

Test Statistics[a,b]

	Evaluation	Activity	Potency
Chi-Square	4.640	21.451	4.112
df	1	1	1
Asymp. Sig.	.031	.000	.043

a. Kruskal Wallis Test

b. Grouping Variable\: A1~A5V1

NPar Tests

Descriptive Statistics

	N	Mean	Std. Deviation	Minimum	Maximum	Percentiles		
						25th	50th (Median)	75th
Evaluation	69	.5589	.29760	-.44	1.00	.4375	.6250	.7500
Activity	69	.1395	.26971	-.56	.63	.0000	.1875	.3750
Potency	69	.4447	.29365	-.38	.88	.3125	.5000	.6875
A1~A5V1	69	30.57	24.581	11	61	11.00	11.00	61.00

Kruskal-Wallis Test

Ranks

	A1~A5V1	N	Mean Rank
Evaluation	A1V1	42	41.11
	V1	27	25.50
	Total	69	
Activity	A1V1	42	43.57
	V1	27	21.67
	Total	69	
Potency	A1V1	42	39.04
	V1	27	28.72
	Total	69	

Test Statistics[a,b]

	Evaluation	Activity	Potency
Chi-Square	10.027	19.744	4.369
df	1	1	1
Asymp. Sig.	.002	.000	.037

a. Kruskal Wallis Test
b. Grouping Variable\: A1~A5V1

NPar Tests

Descriptive Statistics

	N	Mean	Std. Deviation	Minimum	Maximum	Percentiles 25th	50th (Median)	75th
Evaluation	80	.4063	.35786	-.56	.94	.1875	.5000	.6719
Activity	80	.1461	.27755	-.56	.63	-.0469	.1875	.3125
Potency	80	.3016	.36522	-.88	.94	.1250	.3750	.5625
A1~A5V1	80	29.86	22.364	14	61	14.00	14.00	61.00

Kruskal-Wallis Test

Ranks

	A1~A5V1	N	Mean Rank
Evaluation	A1V4	53	41.00
	V1	27	39.52
	Total	80	
Activity	A1V4	53	48.67
	V1	27	24.46
	Total	80	
Potency	A1V4	53	39.23
	V1	27	43.00
	Total	80	

Test Statistics[a,b]

	Evaluation	Activity	Potency
Chi-Square	.073	19.583	.474
df	1	1	1

Asymp. Sig.	.787	.000	.491

a. Kruskal Wallis Test
b. Grouping Variable\: A1~A5V1

NPar Tests

Descriptive Statistics

	N	Mean	Std. Deviation	Minimum	Maximum	Percentiles		
						25th	50th (Median)	75th
Evaluation	69	.3043	.34945	-.56	1.00	.0938	.3125	.5625
Activity	69	.0335	.33616	-.63	.75	-.2500	.0625	.2500
Potency	69	.2292	.33412	-.63	.94	.0000	.2500	.4688
A1~A5V1	69	37.65	19.665	22	62	22.00	22.00	62.00

Kruskal-Wallis Test

Ranks

	A1~A5V1	N	Mean Rank
Evaluation	A2V2	42	41.99
	V2	27	24.13
	Total	69	
Activity	A2V2	42	42.50
	V2	27	23.33
	Total	69	
Potency	A2V2	42	43.33
	V2	27	22.04
	Total	69	

Test Statistics[a,b]

	Evaluation	Activity	Potency

Chi-Square	13.064	15.067	18.592
df	1	1	1
Asymp. Sig.	.000	.000	.000

a. Kruskal Wallis Test
b. Grouping Variable\: A1~A5V1

NPar Tests

Descriptive Statistics

	N	Mean	Std. Deviation	Minimum	Maximum	Percentiles		
						25th	50th (Median)	75th
Evaluation	80	.0711	.33381	-.56	.81	-.1875	.0625	.3125
Activity	80	-.0648	.31595	-.75	.56	-.2969	-.0625	.1875
Potency	80	.0461	.29813	-.63	.75	-.1875	.0625	.2500
A1~A5V1	80	37.49	17.606	25	62	25.00	25.00	62.00

Kruskal-Wallis Test

Ranks

	A1~A5V1	N	Mean Rank
Evaluation	A2V5	53	39.55
	V2	27	42.37
	Total	80	
Activity	A2V5	53	45.01
	V2	27	31.65
	Total	80	
Potency	A2V5	53	41.80
	V2	27	37.94
	Total	80	

Test Statistics[a,b]

	Evaluation	Activity	Potency
Chi-Square	.265	5.943	.496
df	1	1	1
Asymp. Sig.	.607	.015	.481

a. Kruskal Wallis Test
b. Grouping Variable\: A1~A5V1

NPar Tests

Descriptive Statistics

	N	Mean	Std. Deviation	Minimum	Maximum	Percentiles 25th	50th (Median)	75th
Evaluation	69	.2409	.30586	-.44	1.00	.0000	.3125	.4375
Activity	69	.2274	.21194	-.31	.81	.0938	.2500	.3125
Potency	69	.1866	.38040	-.94	.94	-.0313	.2500	.3750
A1~A5V1	69	44.74	14.749	33	63	33.00	33.00	63.00

Kruskal-Wallis Test

Ranks

	A1~A5V1	N	Mean Rank
Evaluation	A3V3	42	33.96
	V3	27	36.61
	Total	69	
Activity	A3V3	42	36.64
	V3	27	32.44
	Total	69	
Potency	A3V3	42	30.82
	V3	27	41.50
	Total	69	

Test Statistics[a,b]

	Evaluation	Activity	Potency
Chi-Square	.288	.732	4.681
df	1	1	1
Asymp. Sig.	.592	.392	.030

a. Kruskal Wallis Test

b. Grouping Variable\: A1~A5V1

NPar Tests

Descriptive Statistics

	N	Mean	Std. Deviation	Minimum	Maximum	Percentiles		
						25th	50th (Median)	75th
Evaluation	69	-.3406	.30156	-1.00	.50	-.5313	-.3750	-.1250
Activity	69	.1078	.29895	-.63	.88	-.0938	.1250	.3125
Potency	69	-.4067	.30555	-.94	.44	-.6250	-.4375	-.2500
A1~A5V1	69	51.83	9.832	44	64	44.00	44.00	64.00

Kruskal-Wallis Test

Ranks

	A1~A5V1	N	Mean Rank
Evaluation	A4V4	42	29.52
	V4	27	43.52
	Total	69	
Activity	A4V4	42	29.51
	V4	27	43.54
	Total	69	
Potency	A4V4	42	29.18
	V4	27	44.06
	Total	69	

Test Statistics[a,b]

	Evaluation	Activity	Potency
Chi-Square	8.052	8.088	9.101
df	1	1	1
Asymp. Sig.	.005	.004	.003

a. Kruskal Wallis Test
b. Grouping Variable\: A1~A5V1

NPar Tests

Descriptive Statistics

	N	Mean	Std. Deviation	Minimum	Maximum	Percentiles		
						25th	50th (Median)	75th
Evaluation	80	-.3125	.31526	-.94	.63	-.5625	-.3438	-.0781
Activity	80	.0102	.30366	-.75	.88	-.1875	.0000	.1875
Potency	80	-.3492	.30517	-.94	.81	-.5469	-.3750	-.2500
A1~A5V1	80	49.43	10.468	42	64	42.00	42.00	64.00

Kruskal-Wallis Test

Ranks

	A1~A5V1	N	Mean Rank
Evaluation	A4V2	53	36.35
	V4	27	48.65
	Total	80	
Activity	A4V2	53	31.79
	V4	27	57.59
	Total	80	
Potency	A4V2	53	37.21

	V4	27	46.96
	Total	80	

Test Statistics[a,b]

	Evaluation	Activity	Potency
Chi-Square	5.034	22.238	3.183
df	1	1	1
Asymp. Sig.	.025	.000	.074

a. Kruskal Wallis Test
b. Grouping Variable\: A1~A5V1

NPar Tests

Descriptive Statistics

	N	Mean	Std. Deviation	Minimum	Maximum	Percentiles		
						25th	50th (Median)	75th
Evaluation	69	-.2011	.35853	-1.00	.56	-.4375	-.1875	.0000
Activity	69	.1884	.26024	-.56	1.00	.0625	.1875	.3750
Potency	68	-.3171	.35476	-1.00	.56	-.5625	-.3750	-.0625
A1~A5V1	69	58.91	4.916	55	65	55.00	55.00	65.00

Kruskal-Wallis Test

Ranks

	A1~A5V1	N	Mean Rank
Evaluation	A5V5	42	35.76
	V5	27	33.81
	Total	69	
Activity	A5V5	42	40.26
	V5	27	26.81

	Total	69	
Potency	A5V5	42	37.13
	V5	26	30.25
	Total	68	

Test Statistics[a,b]

	Evaluation	Activity	Potency
Chi-Square	.155	7.482	1.952
df	1	1	1
Asymp. Sig.	.693	.006	.162

a. Kruskal Wallis Test
b. Grouping Variable\: A1~A5V1

NPar Tests

Descriptive Statistics

	N	Mean	Std. Deviation	Minimum	Maximum	Percentiles 25th	Percentiles 50th (Median)	Percentiles 75th
Evaluation	80	-.3398	.33204	-.88	.56	-.5625	-.3750	-.1875
Activity	80	.1047	.34445	-.69	1.00	-.1250	.1250	.3750
Potency	79	-.3742	.29221	-1.00	.56	-.5000	-.4375	-.2500
A1~A5V1	80	55.72	6.662	51	65	51.00	51.00	65.00

Kruskal-Wallis Test

Ranks

	A1~A5V1	N	Mean Rank
Evaluation	A5V1	53	36.43
	V5	27	48.48
	Total	80	

Activity	A5V1	53	41.40
	V5	27	38.74
	Total	80	
Potency	A5V1	53	41.33
	V5	26	37.29
	Total	79	

Test Statistics[a,b]

	Evaluation	Activity	Potency
Chi-Square	4.836	.235	.546
df	1	1	1
Asymp. Sig.	.028	.628	.460

a. Kruskal Wallis Test

b. Grouping Variable\: A1~A5V1
