# Supplementary material for: Arousal Rules: An Empirical Investigation into the Aesthetic Experience of Cross-Modal Perception with Emotional Visual Music
Source: Front Psychol. 2017 Apr 4;8:440. doi: 10.3389/fpsyg.2017.00440 (PMC5379063; doi:10.3389/fpsyg.2017.00440)
Supplement: Table S3 — One-way ANOVA analysis results for comparing clips within the same modality. [file Table3.PDF]

Audio Only

The evaluation index confirmed that the overall valence of our positive emotion design pieces (A1, A2, and A3) was perceived as a positive affect and the two new audio stimuli (A4 and A5) were perceived as a negative affect (i.e., evaluation index < 0). The A1 piece had the most positive valence, and A5 had the most negative valence according to the evaluation index results. The activity index showed that A3 had high-active arousal levels while A2 had mid-neutral arousal levels (cf., Figure S1). The potency index indicated A1 had highest texture/control levels while A5 had lowest texture/control levels.

One-way ANOVA between Movies in Audio Only

```
ONEWAY evaluation activity potency BY Movies
  /STATISTICS DESCRIPTIVES HOMOGENEITY
  /MISSING ANALYSIS
  /POSTHOC=BONFERRONI ALPHA(0.05).
```

Descriptives

						95% Confidence Interval for Mean		
		N	Mean	Std. Deviation	Std. Error	Lower Bound	Upper Bound	Minimum
evaluation	A1	33	.59470	.249571	.043445	.50620	.68319	-.063
	A2	33	.32765	.210810	.036697	.25290	.40240	-.063
	A3	33	.15909	.230219	.040076	.07746	.24072	-.250
	A4	33	-.30114	.236684	.041201	-.38506	-.21721	-.750
	A5	33	-.37311	.275322	.047927	-.47073	-.27548	-1.000
	Total	165	.08144	.440831	.034319	.01368	.14920	-1.000
activity	A1	33	.29167	.142921	.024879	.24099	.34234	.000
	A2	33	.18182	.216147	.037626	.10518	.25846	-.438
	A3	33	.49621	.201883	.035143	.42463	.56780	-.125
	A4	33	.44886	.217325	.037831	.37180	.52592	-.188
	A5	33	.29356	.153479	.026717	.23914	.34798	-.125
	Total	165	.34242	.219303	.017073	.30871	.37613	-.438
potency	A1	33	.52083	.240882	.041932	.43542	.60625	-.125
	A2	33	.22159	.267042	.046486	.12690	.31628	-.250
	A3	33	.04167	.293428	.051079	-.06238	.14571	-.500
	A4	33	-.50947	.223782	.038955	-.58882	-.43012	-1.000
	A5	33	-.22917	.277169	.048249	-.32745	-.13089	-.750
	Total	165	.00909	.440822	.034318	-.05867	.07685	-1.000

Descriptives

	Maximum

evaluation	A1	1.000
	A2	.875
	A3	.813
	A4	.188
	A5	.250
	Total	1.000
activity	A1	.625
	A2	.438
	A3	.813
	A4	.813
	A5	.500
	Total	.813
potency	A1	.875
	A2	.938
	A3	.750
	A4	-.125
	A5	.438
	Total	.938

Test of Homogeneity of Variances

	Levene Statistic	df1	df2	Sig.
evaluation	.649	4	160	.629
activity	1.709	4	160	.150
potency	.579	4	160	.678

ANOVA

		Sum of Squares	df	Mean Square	F	Sig.
evaluation	Between Groups	22.541	4	5.635	96.643	.000
	Within Groups	9.330	160	.058		
	Total	31.871	164			
activity	Between Groups	2.169	4	.542	15.176	.000
	Within Groups	5.718	160	.036		
	Total	7.887	164			
potency	Between Groups	20.914	4	5.229	76.366	.000
	Within Groups	10.955	160	.068		
	Total	31.869	164			

Post Hoc Tests

Multiple Comparisons

Bonferroni

Dependent Variable	(I) A1~A5V1	(J) A1~A5V1	Mean Difference (I-J)	Std. Error	Sig.	95% Confidence Interval	
						Lower Bound	Upper Bound
evaluation	A1	A2	.267045*	.059447	.000	.09783	.43626
		A3	.435606*	.059447	.000	.26639	.60482
		A4	.895833*	.059447	.000	.72662	1.06505
		A5	.967803*	.059447	.000	.79859	1.13702
	A2	A1	-.267045*	.059447	.000	-.43626	-.09783
		A3	.168561	.059447	.052	-.00065	.33778
		A4	.628788*	.059447	.000	.45957	.79800
		A5	.700758*	.059447	.000	.53154	.86997
	A3	A1	-.435606*	.059447	.000	-.60482	-.26639
		A2	-.168561	.059447	.052	-.33778	.00065
		A4	.460227*	.059447	.000	.29101	.62944
		A5	.532197*	.059447	.000	.36298	.70141
	A4	A1	-.895833*	.059447	.000	-1.06505	-.72662
		A2	-.628788*	.059447	.000	-.79800	-.45957
		A3	-.460227*	.059447	.000	-.62944	-.29101
		A5	.071970	.059447	1.000	-.09724	.24118
	A5	A1	-.967803*	.059447	.000	-1.13702	-.79859
		A2	-.700758*	.059447	.000	-.86997	-.53154
		A3	-.532197*	.059447	.000	-.70141	-.36298
		A4	-.071970	.059447	1.000	-.24118	.09724
activity	A1	A2	.109848	.046539	.195	-.02263	.24232
		A3	-.204545*	.046539	.000	-.33702	-.07207
		A4	-.157197*	.046539	.009	-.28967	-.02472
		A5	-.001894	.046539	1.000	-.13437	.13058
	A2	A1	-.109848	.046539	.195	-.24232	.02263
		A3	-.314394*	.046539	.000	-.44687	-.18192
		A4	-.267045*	.046539	.000	-.39952	-.13457
		A5	-.111742	.046539	.175	-.24422	.02073
	A3	A1	.204545*	.046539	.000	.07207	.33702
		A2	.314394*	.046539	.000	.18192	.44687
		A4	.047348	.046539	1.000	-.08513	.17982
		A5	.202652*	.046539	.000	.07018	.33513
	A4	A1	.157197*	.046539	.009	.02472	.28967
		A2	.267045*	.046539	.000	.13457	.39952
		A3	-.047348	.046539	1.000	-.17982	.08513
		A5	.155303*	.046539	.011	.02283	.28778
	A5	A1	.001894	.046539	1.000	-.13058	.13437
		A2	.111742	.046539	.175	-.02073	.24422
		A3	-.202652*	.046539	.000	-.33513	-.07018
		A4	-.155303*	.046539	.011	-.28778	-.02283

potency	A1	A2	.299242*	.064417	.000	.11588	.48260
		A3	.479167*	.064417	.000	.29580	.66253
		A4	1.030303*	.064417	.000	.84694	1.21366
		A5	.750000*	.064417	.000	.56664	.93336
	A2	A1	-.299242*	.064417	.000	-.48260	-.11588
		A3	.179924	.064417	.059	-.00344	.36329
		A4	.731061*	.064417	.000	.54770	.91442
		A5	.450758*	.064417	.000	.26740	.63412
	A3	A1	-.479167*	.064417	.000	-.66253	-.29580
		A2	-.179924	.064417	.059	-.36329	.00344
		A4	.551136*	.064417	.000	.36777	.73450
		A5	.270833*	.064417	.000	.08747	.45420
	A4	A1	-1.030303*	.064417	.000	-1.21366	-.84694
		A2	-.731061*	.064417	.000	-.91442	-.54770
		A3	-.551136*	.064417	.000	-.73450	-.36777
		A5	-.280303*	.064417	.000	-.46366	-.09694
	A5	A1	-.750000*	.064417	.000	-.93336	-.56664
		A2	-.450758*	.064417	.000	-.63412	-.26740
		A3	-.270833*	.064417	.000	-.45420	-.08747
		A4	.280303*	.064417	.000	.09694	.46366

*. The mean difference is significant at the 0.05 level.

Video Only

Similarly to the audio stimuli, the overall valence of our positive emotion design pieces (V1, V2, and V3) was perceived as a positive affect (i.e. evaluation index ≥ 0), and the negative emotion pieces (V4 and V5) were perceived as a negative affect (i.e. evaluation index ≤ 0). The V1 piece was assessed as the most positive, and V4 and V5 as the most negative in valence levels according to the evaluation index. The activity index indicated that V4 had highest arousal levels while V2 had lowest arousal levels. The potency index indicated V1 had highest texture/control levels while V5 had lowest texture/control levels.

One-way ANOVA between Movies in Visual Only

```
ONEWAY evaluation activity potency BY Movies
  /STATISTICS DESCRIPTIVES HOMOGENEITY
  /MISSING ANALYSIS
  /POSTHOC=BONFERRONI ALPHA(0.05).
```

Descriptives

		N	Mean	Std. Deviation	Std. Error	95% Confidence Interval for Mean Lower Bound	95% Confidence Interval for Mean Upper Bound	Minimum	
evaluation	V1	27	.40046	.350677	.067488	.26174	.53919	-.438	
	V2	27	.10185	.366960	.070622	-.04331	.24702	-.563	
	V3	27	.26389	.310248	.059707	.14116	.38662	-.375	
	V4	27	-.21296	.292257	.056245	-.32858	-.09735	-.813	
	V5	27	-.20139	.404799	.077904	-.36152	-.04126	-.813	
	Total	135	.07037	.421650	.036290	-.00140	.14215	-.813	
activity	V1	27	-.05093	.302750	.058264	-.17069	.06884	-.563	
	V2	27	-.17361	.350709	.067494	-.31235	-.03488	-.625	
	V3	27	.21065	.208120	.040053	.12832	.29298	-.188	
	V4	27	.23611	.303888	.058483	.11590	.35633	-.500	
	V5	27	.08796	.348097	.066991	-.04974	.22567	-.563	
	Total	135	.06204	.340491	.029305	.00408	.12000	-.625	
potency	V1	27	.34954	.338912	.065224	.21547	.48361	-.375	
	V2	27	.00463	.325188	.062582	-.12401	.13327	-.625	
	V3	27	.30787	.366049	.070446	.16307	.45267	-.625	
	V4	27	-.28935	.267505	.051481	-.39517	-.18353	-.688	
	V5	27	-.40972	.384235	.073946	-.56172	-.25772	-1.000	
	Total	135	-.00741	.453534	.039034	-.08461	.06980	-1.000	

Descriptives

		Maximum
evaluation	V1	.938
	V2	.813
	V3	1.000
	V4	.500
	V5	.563
	Total	1.000
activity	V1	.625
	V2	.563
	V3	.813
	V4	.875
	V5	1.000
	Total	1.000
potency	V1	.875
	V2	.625
	V3	.938
	V4	.375
	V5	.563
	Total	.938

Test of Homogeneity of Variances

	Levene Statistic	df1	df2	Sig.
evaluation	1.150	4	130	.336
activity	2.217	4	130	.071
potency	.617	4	130	.651

ANOVA

		Sum of Squares	df	Mean Square	F	Sig.
evaluation	Between Groups	8.141	4	2.035	16.872	.000
	Within Groups	15.682	130	.121		
	Total	23.824	134			
activity	Between Groups	3.276	4	.819	8.686	.000
	Within Groups	12.259	130	.094		
	Total	15.535	134			
potency	Between Groups	12.644	4	3.161	27.545	.000
	Within Groups	14.919	130	.115		
	Total	27.563	134			

Post Hoc Tests

Multiple Comparisons

Bonferroni

Dependent Variable	(I) A1~A5V1	(J) A1~A5V1	Mean Difference (I-J)	Std. Error	Sig.	95% Confidence Interval	
						Lower Bound	Upper Bound
evaluation	V1	V2	.298611*	.094529	.020	.02866	.56856
		V3	.136574	.094529	1.000	-.13338	.40652
		V4	.613426*	.094529	.000	.34348	.88338
		V5	.601852*	.094529	.000	.33190	.87180
	V2	V1	-.298611*	.094529	.020	-.56856	-.02866
		V3	-.162037	.094529	.889	-.43199	.10791
		V4	.314815*	.094529	.011	.04486	.58477
		V5	.303241*	.094529	.017	.03329	.57319
	V3	V1	-.136574	.094529	1.000	-.40652	.13338
		V2	.162037	.094529	.889	-.10791	.43199
		V4	.476852*	.094529	.000	.20690	.74680
		V5	.465278*	.094529	.000	.19533	.73523
	V4	V1	-.613426*	.094529	.000	-.88338	-.34348
		V2	-.314815*	.094529	.011	-.58477	-.04486
		V3	-.476852*	.094529	.000	-.74680	-.20690
		V5	-.011574	.094529	1.000	-.28152	.25838
	V5	V1	-.601852*	.094529	.000	-.87180	-.33190
		V2	-.303241*	.094529	.017	-.57319	-.03329
		V3	-.465278*	.094529	.000	-.73523	-.19533
		V4	.011574	.094529	1.000	-.25838	.28152
activity	V1	V2	.122685	.083576	1.000	-.11599	.36136
		V3	-.261574*	.083576	.022	-.50025	-.02290
		V4	-.287037*	.083576	.008	-.52571	-.04837
		V5	-.138889	.083576	.990	-.37756	.09978
	V2	V1	-.122685	.083576	1.000	-.36136	.11599
		V3	-.384259*	.083576	.000	-.62293	-.14559
		V4	-.409722*	.083576	.000	-.64839	-.17105
		V5	-.261574*	.083576	.022	-.50025	-.02290
	V3	V1	.261574*	.083576	.022	.02290	.50025
		V2	.384259*	.083576	.000	.14559	.62293
		V4	-.025463	.083576	1.000	-.26413	.21321
		V5	.122685	.083576	1.000	-.11599	.36136
	V4	V1	.287037*	.083576	.008	.04837	.52571
		V2	.409722*	.083576	.000	.17105	.64839
		V3	.025463	.083576	1.000	-.21321	.26413
		V5	.148148	.083576	.786	-.09052	.38682
	V5	V1	.138889	.083576	.990	-.09978	.37756
		V2	.261574*	.083576	.022	.02290	.50025
		V3	-.122685	.083576	1.000	-.36136	.11599
		V4	-.148148	.083576	.786	-.38682	.09052

potency	V1	V2	.344907*	.092199	.003	.08161	.60820
		V3	.041667	.092199	1.000	-.22163	.30496
		V4	.638889*	.092199	.000	.37559	.90219
		V5	.759259*	.092199	.000	.49596	1.02256
	V2	V1	-.344907*	.092199	.003	-.60820	-.08161
		V3	-.303241*	.092199	.013	-.56654	-.03994
		V4	.293981*	.092199	.018	.03069	.55728
		V5	.414352*	.092199	.000	.15106	.67765
	V3	V1	-.041667	.092199	1.000	-.30496	.22163
		V2	.303241*	.092199	.013	.03994	.56654
		V4	.597222*	.092199	.000	.33393	.86052
		V5	.717593*	.092199	.000	.45430	.98089
	V4	V1	-.638889*	.092199	.000	-.90219	-.37559
		V2	-.293981*	.092199	.018	-.55728	-.03069
		V3	-.597222*	.092199	.000	-.86052	-.33393
		V5	.120370	.092199	1.000	-.14293	.38367
	V5	V1	-.759259*	.092199	.000	-1.02256	-.49596
		V2	-.414352*	.092199	.000	-.67765	-.15106
		V3	-.717593*	.092199	.000	-.98089	-.45430
		V4	-.120370	.092199	1.000	-.38367	.14293

*. The mean difference is significant at the 0.05 level.

Original Visual Music

In original visual music group, similarly as audio-only and video-only, our positive emotion design pieces (A1V1, A2V2, and A3V3) were perceived as a positive affect and the two new audio stimuli (A4V4 and A5V5) were perceived as a negative affect (i.e., evaluation index < 0). The A1V1 piece was assessed as the most positive visual music in valence, and A4V4 had the most negative valence according to the evaluation index results. The activity index showed that A1V1 had high-active arousal levels while A4V4 had neutral arousal levels. The potency index indicated A1V1 had highest texture/control levels while A4V4 had lowest arousal levels.

One-way ANOVA between Movies in Original Visual Music

```
ONEWAY evaluation activity potency BY MovieID
  /STATISTICS DESCRIPTIVES HOMOGENEITY
  /MISSING ANALYSIS
  /POSTHOC=BONFERRONI ALPHA(0.05).
```

Descriptives

		N	Mean	Std. Deviation	Std. Error	95% Confidence Interval for Mean		Minimum	
						Lower Bound	Upper Bound		
evaluation	A1V1	42	.66071	.204346	.031531	.59704	.72439	.125	
	A2V2	42	.43452	.269779	.041628	.35045	.51859	-.125	
	A3V3	42	.22619	.305860	.047195	.13088	.32150	-.438	
	A4V4	42	-.42262	.281143	.043381	-.51023	-.33501	-1.000	
	A5V5	42	-.20089	.330585	.051010	-.30391	-.09788	-1.000	
	Total	210	.13958	.487998	.033675	.07320	.20597	-1.000	
activity	A1V1	42	.26190	.152618	.023550	.21435	.30946	-.063	
	A2V2	42	.16667	.251014	.038732	.08845	.24489	-.500	
	A3V3	42	.23810	.216171	.033356	.17073	.30546	-.313	
	A4V4	42	.02530	.268045	.041360	-.05823	.10883	-.625	
	A5V5	42	.25298	.156754	.024188	.20413	.30182	-.063	
	Total	210	.18899	.229940	.015867	.15771	.22027	-.625	
potency	A1V1	42	.50595	.245698	.037912	.42939	.58252	-.250	
	A2V2	42	.37351	.252083	.038897	.29496	.45207	-.125	
	A3V3	42	.10863	.372977	.057552	-.00760	.22486	-.938	
	A4V4	42	-.48214	.307517	.047451	-.57797	-.38631	-.938	
	A5V5	42	-.27381	.340359	.052519	-.37987	-.16775	-1.000	
	Total	210	.04643	.484087	.033405	-.01943	.11228	-1.000	

Descriptives

		Maximum
evaluation	A1V1	1.000
	A2V2	1.000
	A3V3	.938
	A4V4	.188
	A5V5	.500
	Total	1.000
activity	A1V1	.500
	A2V2	.750
	A3V3	.750
	A4V4	.500
	A5V5	.563
	Total	.750
potency	A1V1	.875
	A2V2	.938
	A3V3	.938
	A4V4	.438
	A5V5	.500
	Total	.938

Test of Homogeneity of Variances

	Levene Statistic	df1	df2	Sig.
evaluation	1.480	4	205	.210
activity	3.225	4	205	.014
potency	2.924	4	205	.022

ANOVA

		Sum of Squares	df	Mean Square	F	Sig.
evaluation	Between Groups	33.519	4	8.380	105.693	.000
	Within Groups	16.253	205	.079		
	Total	49.772	209			
activity	Between Groups	1.643	4	.411	8.950	.000
	Within Groups	9.407	205	.046		
	Total	11.050	209			
potency	Between Groups	29.566	4	7.392	78.062	.000
	Within Groups	19.411	205	.095		
	Total	48.977	209			

Post Hoc Tests

Multiple Comparisons

Bonferroni

Dependent Variable	(I) A1~A5V1	(J) A1~A5V1	Mean Difference (I-J)	Std. Error	Sig.	95% Confidence Interval	
						Lower Bound	Upper Bound
evaluation	A1V1	A2V2	.226190*	.061444	.003	.05183	.40055
		A3V3	.434524*	.061444	.000	.26016	.60889
		A4V4	1.083333*	.061444	.000	.90897	1.25770
		A5V5	.861607*	.061444	.000	.68724	1.03597
	A2V2	A1V1	-.226190*	.061444	.003	-.40055	-.05183
		A3V3	.208333*	.061444	.008	.03397	.38270
		A4V4	.857143*	.061444	.000	.68278	1.03151
		A5V5	.635417*	.061444	.000	.46105	.80978
	A3V3	A1V1	-.434524*	.061444	.000	-.60889	-.26016
		A2V2	-.208333*	.061444	.008	-.38270	-.03397
		A4V4	.648810*	.061444	.000	.47445	.82317
		A5V5	.427083*	.061444	.000	.25272	.60145
	A4V4	A1V1	-1.083333*	.061444	.000	-1.25770	-.90897
		A2V2	-.857143*	.061444	.000	-1.03151	-.68278
		A3V3	-.648810*	.061444	.000	-.82317	-.47445
		A5V5	-.221726*	.061444	.004	-.39609	-.04736
	A5V5	A1V1	-.861607*	.061444	.000	-1.03597	-.68724
		A2V2	-.635417*	.061444	.000	-.80978	-.46105
		A3V3	-.427083*	.061444	.000	-.60145	-.25272
		A4V4	.221726*	.061444	.004	.04736	.39609
activity	A1V1	A2V2	.095238	.046747	.429	-.03742	.22789
		A3V3	.023810	.046747	1.000	-.10884	.15646
		A4V4	.236607*	.046747	.000	.10395	.36926
		A5V5	.008929	.046747	1.000	-.12373	.14158
	A2V2	A1V1	-.095238	.046747	.429	-.22789	.03742
		A3V3	-.071429	.046747	1.000	-.20408	.06123
		A4V4	.141369*	.046747	.028	.00871	.27402
		A5V5	-.086310	.046747	.663	-.21896	.04634
	A3V3	A1V1	-.023810	.046747	1.000	-.15646	.10884
		A2V2	.071429	.046747	1.000	-.06123	.20408
		A4V4	.212798*	.046747	.000	.08014	.34545
		A5V5	-.014881	.046747	1.000	-.14754	.11777
	A4V4	A1V1	-.236607*	.046747	.000	-.36926	-.10395
		A2V2	-.141369*	.046747	.028	-.27402	-.00871
		A3V3	-.212798*	.046747	.000	-.34545	-.08014
		A5V5	-.227679*	.046747	.000	-.36033	-.09502
	A5V5	A1V1	-.008929	.046747	1.000	-.14158	.12373
		A2V2	.086310	.046747	.663	-.04634	.21896
		A3V3	.014881	.046747	1.000	-.11777	.14754

		A4V4	.227679*	.046747	.000	.09502	.36033
potency	A1V1	A2V2	.132440	.067148	.499	-.05811	.32299
		A3V3	.397321*	.067148	.000	.20677	.58787
		A4V4	.988095*	.067148	.000	.79755	1.17865
		A5V5	.779762*	.067148	.000	.58921	.97031
	A2V2	A1V1	-.132440	.067148	.499	-.32299	.05811
		A3V3	.264881*	.067148	.001	.07433	.45543
		A4V4	.855655*	.067148	.000	.66510	1.04620
		A5V5	.647321*	.067148	.000	.45677	.83787
	A3V3	A1V1	-.397321*	.067148	.000	-.58787	-.20677
		A2V2	-.264881*	.067148	.001	-.45543	-.07433
		A4V4	.590774*	.067148	.000	.40022	.78132
		A5V5	.382440*	.067148	.000	.19189	.57299
	A4V4	A1V1	-.988095*	.067148	.000	-1.17865	-.79755
		A2V2	-.855655*	.067148	.000	-1.04620	-.66510
		A3V3	-.590774*	.067148	.000	-.78132	-.40022
		A5V5	-.208333*	.067148	.022	-.39888	-.01778
	A5V5	A1V1	-.779762*	.067148	.000	-.97031	-.58921
		A2V2	-.647321*	.067148	.000	-.83787	-.45677
		A3V3	-.382440*	.067148	.000	-.57299	-.19189
		A4V4	.208333*	.067148	.022	.01778	.39888

*. The mean difference is significant at the 0.05 level.

Altered Visual Music

For altered visual music mode, evaluation assessment indicated that most of the pairs as significantly different ($p<0.001$, Bonferroni correction), excepted for the pair A2V5-A3V3 ($p = 0.105$), and A4V4-A5V1 ($p=1.00$). The activity index returned 3 pairs to be significantly distinct (all pairs, Bonferroni correction): A1V4-A3V3 ($p = 1.00$), A1V4-A5V1 ($p = 0.130$), and A2V5- A4V2 ($p < 0.741$). For potency, all comparisons returned significant distinctions ($p<0.001$) except 1 pairs A1V4-A3V3 ($p=1.000$), and A2V5-A3V3 ($p=0.694$), A3V3-A5V1 ($p=0.101$).

One-way ANOVA between Movies in Altered Visual Music

```
ONEWAY evaluation activity potency BY MovieID
  /STATISTICS DESCRIPTIVES HOMOGENEITY
  /MISSING ANALYSIS
  /POSTHOC=BONFERRONI ALPHA(0.05).
```

Descriptives

		N	Mean	Std. Deviation	Std. Error	95% Confidence Interval for Mean Lower Bound	95% Confidence Interval for Mean Upper Bound	Minimum
evaluation	A1V4	53	.40920	.364757	.050103	.30866	.50974	-.563
	A2V5	53	.05542	.318137	.043699	-.03226	.14311	-.563
	A3V3	53	.21226	.291697	.040068	.13186	.29267	-.500
	A4V2	53	-.36321	.317048	.043550	-.45060	-.27582	-.938
	A5V1	53	-.41038	.265592	.036482	-.48358	-.33717	-.875
	Total	265	-.01934	.446979	.027458	-.07340	.03472	-.938
activity	A1V4	53	.24646	.201934	.027738	.19080	.30212	-.500
	A2V5	53	-.00943	.284410	.039067	-.08783	.06896	-.750
	A3V3	53	.25118	.284435	.039070	.17278	.32958	-.625
	A4V2	53	-.10495	.232169	.031891	-.16895	-.04096	-.750
	A5V1	53	.11321	.345615	.047474	.01794	.20847	-.688
	Total	265	.09929	.306287	.018815	.06225	.13634	-.750
potency	A1V4	53	.27712	.378690	.052017	.17274	.38150	-.875
	A2V5	53	.06722	.284265	.039047	-.01114	.14557	-.563
	A3V3	53	.17925	.334791	.045987	.08697	.27153	-.625
	A4V2	53	-.37972	.320768	.044061	-.46813	-.29130	-.938
	A5V1	53	-.36792	.247178	.033952	-.43606	-.29979	-1.000
	Total	265	-.04481	.418835	.025729	-.09547	.00585	-1.000

Descriptives

		Maximum
evaluation	A1V4	.938
	A2V5	.750
	A3V3	.875
	A4V2	.625
	A5V1	.188
	Total	.938
activity	A1V4	.563
	A2V5	.500
	A3V3	1.000
	A4V2	.438
	A5V1	.813
	Total	1.000
potency	A1V4	.938
	A2V5	.750
	A3V3	.813
	A4V2	.813
	A5V1	.125
	Total	.938

Test of Homogeneity of Variances

	Levene Statistic	df1	df2	Sig.
evaluation	.572	4	260	.683
activity	5.761	4	260	.000
potency	2.413	4	260	.050

ANOVA

		Sum of Squares	df	Mean Square	F	Sig.
evaluation	Between Groups	27.244	4	6.811	69.442	.000
	Within Groups	25.501	260	.098		
	Total	52.745	264			
activity	Between Groups	5.218	4	1.305	17.352	.000
	Within Groups	19.548	260	.075		
	Total	24.766	264			
potency	Between Groups	20.297	4	5.074	50.713	.000
	Within Groups	26.015	260	.100		
	Total	46.312	264			

Post Hoc Tests

Multiple Comparisons

Bonferroni

Dependent Variable	(I) A1~A5V1	(J) A1~A5V1	Mean Difference (I-J)	Std. Error	Sig.	95% Confidence Interval	
						Lower Bound	Upper Bound
evaluation	A1V4	A2V5	.353774*	.060837	.000	.18153	.52602
		A3V3	.196934*	.060837	.014	.02469	.36918
		A4V2	.772406*	.060837	.000	.60016	.94465
		A5V1	.819575*	.060837	.000	.64733	.99182
	A2V5	A1V4	-.353774*	.060837	.000	-.52602	-.18153
		A3V3	-.156840	.060837	.105	-.32908	.01540
		A4V2	.418632*	.060837	.000	.24639	.59087
		A5V1	.465802*	.060837	.000	.29356	.63804
	A3V3	A1V4	-.196934*	.060837	.014	-.36918	-.02469
		A2V5	.156840	.060837	.105	-.01540	.32908
		A4V2	.575472*	.060837	.000	.40323	.74771
		A5V1	.622642*	.060837	.000	.45040	.79488
	A4V2	A1V4	-.772406*	.060837	.000	-.94465	-.60016
		A2V5	-.418632*	.060837	.000	-.59087	-.24639
		A3V3	-.575472*	.060837	.000	-.74771	-.40323
		A5V1	.047170	.060837	1.000	-.12507	.21941
	A5V1	A1V4	-.819575*	.060837	.000	-.99182	-.64733
		A2V5	-.465802*	.060837	.000	-.63804	-.29356
		A3V3	-.622642*	.060837	.000	-.79488	-.45040
		A4V2	-.047170	.060837	1.000	-.21941	.12507
activity	A1V4	A2V5	.255896*	.053265	.000	.10509	.40670
		A3V3	-.004717	.053265	1.000	-.15552	.14609
		A4V2	.351415*	.053265	.000	.20061	.50222
		A5V1	.133255	.053265	.130	-.01755	.28406
	A2V5	A1V4	-.255896*	.053265	.000	-.40670	-.10509
		A3V3	-.260613*	.053265	.000	-.41142	-.10981
		A4V2	.095519	.053265	.741	-.05528	.24632
		A5V1	-.122642	.053265	.221	-.27344	.02816
	A3V3	A1V4	.004717	.053265	1.000	-.14609	.15552
		A2V5	.260613*	.053265	.000	.10981	.41142
		A4V2	.356132*	.053265	.000	.20533	.50693
		A5V1	.137972	.053265	.101	-.01283	.28877
	A4V2	A1V4	-.351415*	.053265	.000	-.50222	-.20061
		A2V5	-.095519	.053265	.741	-.24632	.05528
		A3V3	-.356132*	.053265	.000	-.50693	-.20533
		A5V1	-.218160*	.053265	.001	-.36896	-.06736
	A5V1	A1V4	-.133255	.053265	.130	-.28406	.01755
		A2V5	.122642	.053265	.221	-.02816	.27344
		A3V3	-.137972	.053265	.101	-.28877	.01283

		A4V2	.218160*	.053265	.001	.06736	.36896
potency	A1V4	A2V5	.209906*	.061447	.007	.03594	.38387
		A3V3	.097877	.061447	1.000	-.07609	.27185
		A4V2	.656840*	.061447	.000	.48287	.83081
		A5V1	.645047*	.061447	.000	.47108	.81902
	A2V5	A1V4	-.209906*	.061447	.007	-.38387	-.03594
		A3V3	-.112028	.061447	.694	-.28600	.06194
		A4V2	.446934*	.061447	.000	.27297	.62090
		A5V1	.435142*	.061447	.000	.26117	.60911
	A3V3	A1V4	-.097877	.061447	1.000	-.27185	.07609
		A2V5	.112028	.061447	.694	-.06194	.28600
		A4V2	.558962*	.061447	.000	.38499	.73293
		A5V1	.547170*	.061447	.000	.37320	.72114
	A4V2	A1V4	-.656840*	.061447	.000	-.83081	-.48287
		A2V5	-.446934*	.061447	.000	-.62090	-.27297
		A3V3	-.558962*	.061447	.000	-.73293	-.38499
		A5V1	-.011792	.061447	1.000	-.18576	.16218
	A5V1	A1V4	-.645047*	.061447	.000	-.81902	-.47108
		A2V5	-.435142*	.061447	.000	-.60911	-.26117
		A3V3	-.547170*	.061447	.000	-.72114	-.37320
		A4V2	.011792	.061447	1.000	-.16218	.18576

*. The mean difference is significant at the 0.05 level.
